# Endothelial dysfunction contributes to severe COVID-19 in combination with dysregulated lymphocyte responses and cytokine networks

**DOI:** 10.1038/s41392-021-00819-6

**Published:** 2021-12-10

**Authors:** Louisa Ruhl, Isabell Pink, Jenny F. Kühne, Kerstin Beushausen, Jana Keil, Stella Christoph, Andrea Sauer, Lennart Boblitz, Julius Schmidt, Sascha David, Hans-Martin Jäck, Edith Roth, Markus Cornberg, Thomas F. Schulz, Tobias Welte, Marius M. Höper, Christine S. Falk

**Affiliations:** 1 Institute of Transplant Immunology, MHH, D, Hanover, Germany; 2 Department of Pneumology, MHH, D, Hanover, Germany; 3 Department of Intensive Care Medicine, University of Zurich, CH, Zurich, Switzerland; 4 Division of Molecular Immunology, Internal Medicine III, Nikolaus-Fiebiger-Center of Molecular Medicine, Friedrich-Alexander University Erlangen-Nürnberg, D, Erlangen, Germany; 5 Department of Gastroenterology Hepatology and Endocrinology, MHH, D, Hanover, Germany; 6 Center for Individualized Infection Medicine CiiM, Hanover, Germany; 7 Institute of Virology, MHH, D, Hanover, Germany; 8 German Center for Infection Research, DZIF, TTU-IICH, Marburg, Germany; 9 Excellence Cluster 2155 RESIST, MHH, Hanover, Germany; 10 German Center for Lung Diseases DZL/BREATH, Marburg, Germany

**Keywords:** Adaptive immunity, Infectious diseases

## Abstract

The systemic processes involved in the manifestation of life-threatening COVID-19 and in disease recovery are still incompletely understood, despite investigations focusing on the dysregulation of immune responses after SARS-CoV-2 infection. To define hallmarks of severe COVID-19 in acute disease (*n* = 58) and in disease recovery in convalescent patients (*n* = 28) from Hannover Medical School, we used flow cytometry and proteomics data with unsupervised clustering analyses. In our observational study, we combined analyses of immune cells and cytokine/chemokine networks with endothelial activation and injury. ICU patients displayed an altered immune signature with prolonged lymphopenia but the expansion of granulocytes and plasmablasts along with activated and terminally differentiated T and NK cells and high levels of SARS-CoV-2-specific antibodies. The core signature of seven plasma proteins revealed a highly inflammatory microenvironment in addition to endothelial injury in severe COVID-19. Changes within this signature were associated with either disease progression or recovery. In summary, our data suggest that besides a strong inflammatory response, severe COVID-19 is driven by endothelial activation and barrier disruption, whereby recovery depends on the regeneration of the endothelial integrity.

## Introduction

In December 2019, first cases of patients suffering from severe acute respiratory syndrome (SARS) were observed following infection with the severe acute respiratory syndrome coronavirus 2 (SARS-CoV-2). The new coronavirus rapidly spread around the globe and was declared as a pandemic by the World Health Organization (WHO) on March 11, 2020.^1^ The associated coronavirus disease 2019 (COVID-19) ranges from an asymptomatic state or mild symptoms to severe progression and lethal outcome.^2^ As of May 2021 SARS-CoV-2 infected more than 150,000,000 people worldwide and caused more than 3,200,000 deaths.

The upper respiratory tract and the lung are the primary sites of SARS-CoV-2 infection and dyspnea is a leading symptom of severe COVID-19 with respiratory failure causing admission to intensive care unit (ICU) and mechanical ventilation.^3^ Pulmonary symptoms are mainly caused by infection of epithelial cells, particularly type II alveolar epithelial cells, via angiotensin-converting enzyme 2 (ACE2) as a receptor for the SARS-CoV-2 spike protein following proteolytic cleavage by the TMPRSS2 protease, facilitating viral entry.^4^ Sustained uncontrolled infection results in extensive death of barrier cells within the alveoli and leads to vascular leakage along with tissue edema and activation of coagulation pathways.^5^ These processes ultimately promote the formation of an acute respiratory distress syndrome (ARDS), a major clinical feature of severe COVID-19. Pulmonary complications resulting from endothelial damage can be caused by multiple mechanisms in the case of SARS-CoV-2. Next to infection of endothelial cells by the virus, also decreased activity of ACE2 due to binding of SAS-CoV-2 inducing the kallikrein-bradykinin pathway, can lead to increased barrier breach.^5^ Furthermore, reactive oxygen species produced by activated neutrophils can damage barrier cells and immune cells in addition to inflammatory plasma proteins, which promote endothelial disruption.^5^ In severe cases, patients can also develop further endothelium-associated complications like heart diseases, abnormal blood coagulation, and neurological complications as well as liver and kidney injuries, which can progress to multiorgan failure.^6^ These clinical symptoms indicate crucial participation of the endothelial system in the manifestation of severe COVID-19.

The reasons for the wide range of COVID-19 symptoms and variable, unpredictable disease progressions are under intense investigation. Age and age-related chronic inflammatory conditions such as diabetes together with genetic predispositions represent major risk factors for severe COVID-19.^7^ Furthermore, several studies suggested a direct connection between the host immune response and critical COVID-19, demonstrating dynamic and highly heterogeneous immune signatures with altered immune cell compositions and cytokine/chemokine patterns in severe COVID-19.^8–10^ SARS-CoV-2 seems to manipulate the host immunity by evading especially the innate immune response, as ineffective IFN immunity was associated with fatal COVID-19.^7,11^ Besides the innate, also the adaptive immunity contributes to progression toward severe COVID-19.^11^ In particular, SARS-CoV-2-specific T cells are crucial for rapid virus clearance since an extended absence of virus-specific T cells was related to severe COVID-19 progression.^12^ Regarding the humoral response, >90% of infected individuals seroconverted to SARS-CoV-2 spike (S) and/or nucleocapsid (N) proteins, but rather limited somatic hypermutation was observed for neutralizing antibodies,^9,13^ indicating also an altered B-cell response. Additionally, cytokine profiles from severe SARS-CoV-2 infections revealed a highly inflammatory microenvironment with elevated levels of cytokines like IL-6 and TNF-α and chemokines like CXCL-10 in addition to altered antiviral IFN responses.^7,8,10^

As clinical symptoms indicate a crucial endothelial contribution to severe COVID-19 our study aimed to investigate a potential link between the endothelium and hyperinflammation in critical cases. We focused on identifying immune and endothelial signatures as main drivers for severe COVID-19 progression since uncovering pathological mechanisms may be a first step for developing novel treatment strategies and could help to understand long-lasting effects in recovered patients.^14^ Therefore, we included blood samples from acute severe COVID-19 patients admitted to intensive care unit (ICU, *n* = 58) and from convalescent, former ICU patients (CONV, *n* = 28) in our study. Cellular, humoral, and endothelial responses were analyzed, and patterns were identified by unsupervised clustering and correlation matrices. First, we observed a dominating hyperactivated HLA-DR^+^/CD38^+^ phenotype of memory T and NK cells despite massive T-cell lymphopenia. Second, granulocytes were significantly expanded as well as plasmablasts, which were accompanied by a rapid production of S- and N-specific IgM, IgA, and IgG antibodies. Third, in severe COVID-19, this hyperactivation at the cellular level was accompanied by a highly pro-inflammatory cytokine/chemokine network, which was associated with endothelial activation leading to a stepwise disruption of the endothelial barrier. Finally, unsupervised cluster and correlation analyses identified a unique core chemokine/endothelial signature for severe COVID-19 as well as distinct cytokine signatures associated with recovery from severe disease.

## Results

### Dynamic changes in the entire immune cell composition in severe COVID-19 patients with memory T-cell development and plasmablast expansion

**Fig. 1:**
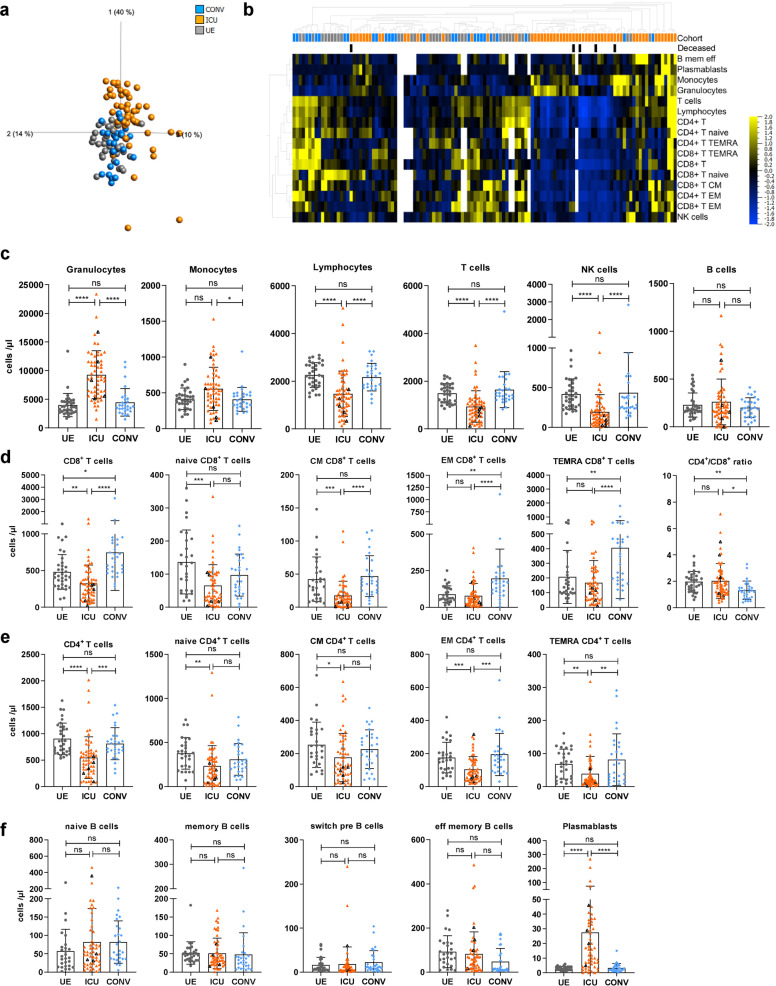
Dynamic changes in the entire immune cell composition in severe COVID-19 patients with memory T-cell development and plasmablast expansion. Immune cell distribution reflected by absolute numbers in patient blood was analyzed using TruCount analyses. **a**, **b** Multigroup comparison with a *P* value cutoff of 0.041 was used to identify significant differences among UE (*n* = 29), ICU (*n* = 58) and CONV (*n* = 28) for (**a**) principal component analysis and (**b**) heatmap analysis. Blue to yellow scale represents the expression values normalized to mean = 0, var = 1. Missing values are displayed in white. **c**–**f** Numbers of different immune cells in patient blood. T cells: naive (CCR7 ^+^ CD45RO^−^), central memory (CM, CCR7 + CD45RO + ), effector memory (EM, CCR7^−^CD45RO ^+^ ) and TEMRA (CCR7^−^CD45RO^−^); B cells: naive (IgD ^+^ CD27^−^) memory (mem, CD27 ^+^ IgD^−^), switch precursor (switch pre, CD27 ^+^ IgD ^+^ ), effector memory (eff mem, IgD^−^CD27^−^) and plasmablasts (CD19 ^+^ CD20^−^CD27 ^+^ CD38 ^+^ ). Black triangles represent last samples from deceased patients. UE: unexposed donors, ICU: intensive care unit patients, CONV: convalescent patients, CM: central memory, EM: effector memory, mem eff: memory effector, switch pre: switch precursor. Statistical analysis: ANOVA test with Turkey multiple comparison test or Kruskal–Wallis with test with Dunn’s multiple comparison test were performed. *P < 0.05, **P < 0.01, ***P < 0.001, ****P < 0.0001

A total of *N* = 42 patients with *n* = 86 samples from MHH with confirmed SARS-CoV-2 infection were analyzed at various time points after symptom onset. *N* = 25 patients (*n* = 58 samples) have been admitted to ICU and *N* = 17 patients (*n* = 28 samples) were convalescent former ICU patients (CONV). *N* = 29 unexposed individuals (UE, *n* = 29 samples) served as control group (S1, Supplementary Table 1). To analyze the leukocyte dynamics during COVID-19, we quantified their absolute numbers and proportions in ICU, CONV, and UE. Unsupervised cluster analyses of our immune phenotyping datasets (Supplementary Fig. S2) revealed a clear separation of ICU from CONV and UE, indicating significant dynamic changes in the lymphocyte compartment during acute COVID-19 (Fig. 1a, b and Supplementary Fig. S3a). As known from other studies,^8,15^ both absolute numbers as well as frequencies of granulocytes were highly elevated in blood of ICU patients while monocytes were stable and only proportionally reduced (Fig. 1c and Supplementary Fig. S3b). In contrast, absolute numbers as well as frequencies of lymphocytes, especially T cells, were significantly decreased in ICU compared to CONV and UE, with some patients showing strong lymphopenia (<1000 lymphocytes/µl blood). The number of NK cells was also decreased, whereas B-cell numbers were unchanged in ICU compared to CONV and UE. Leukocyte numbers and frequencies of CONV displayed no significant differences compared to UE (Fig. 1c and Supplementary Fig. S3b). Interestingly, four out of five deceased ICU patients clustered close to each other and exhibited decreased numbers of T cells and an activated immune phenotype (Fig. 1b and Supplementary Fig. S3a). As reported earlier, ICU patients show a strong decline in lymphocyte numbers, including T and NK cells, leading to lymphopenia along with an unusual expansion of granulocytes. Furthermore, comparable leukocyte numbers and frequencies of CONV and UE indicate recovery at the cellular level in the periphery of CONV.

To further investigate the T-cell phenotypes in severe COVID-19, the distribution of naive vs. memory CD4^+^ and CD8^+^ T cells was determined for ICU, CONV, and UE based on the expression of the chemokine receptor CCR7 and the CD45RO memory marker (Supplementary Fig. S2a). Especially ICU showed highly variable numbers and proportions of all T-cell subsets (Fig. 1d, e and Supplementary Fig. S3c, d). CD8^+^ T-cell numbers, particularly CCR7^+^CD45RO^−^ naive and CCR7^+^CD45RO^+^ central memory (CM) T cells were decreased in ICU compared to CONV and UE (Fig. 1d). In addition, all CD4^+^ T cells subsets were significantly reduced in numbers (Fig. 1e). With regards to CONV, we found elevated numbers of CD8^+^ T cells compared to UE, especially CCR7^−^CD45RO^+^ effector memory (EM) and CCR7^−^CD45RO^−^ TEMRA at the cost of naive CD8^+^ T cells, which translated into a reduced CD4^+^/CD8^+^ ratio compared to UE and ICU (Fig. 1d). Thus, CD4^+^ and CD8^+^ T cells were both affected by the lymphopenia in COVID-19 ICU patients, and particularly elevated CD8^+^ TEMRA T-cell numbers in CONV suggest a pronounced T-cell differentiation during SARS-CoV-2 infection.

In contrast to T cells, B cells were less affected by lymphopenia in ICU (Fig. 1c). Detailed analyses of B-cell subsets showed a broad range of IgD^+^CD27^−^ naive, CD27^+^IgD^−^ memory (mem), CD27^+^IgD^+^ switch precursor (switch pre), and IgD^−^CD27^–^ effector memory (eff-mem) B cells in ICU accompanied by an unusual expansion of CD19^+^CD20^−^CD27^+^CD38^+^ plasmablasts (Fig. 1f and Supplementary Figs. S2c, S3e). Even though we did not observe significant differences in absolute numbers of these B-cell subsets in ICU, frequencies of naive B cells were increased and frequencies of eff-mem B cells were decreased in CONV compared to UE and ICU (Fig. 1f and Supplementary Fig. S3e). Concluding, elevated absolute numbers and proportions of plasmablasts in ICU patients indicate a strong systemic B-cell response during acute SARS-CoV-2 infection followed by a reverse transformation of the B-cell population in CONV with elevated proportions of naive B cells and reduced proportions of eff-mem B cells.

### Plasmablasts contribute to antibody development and blocking activity is associated with disease severity

**Fig. 2:**
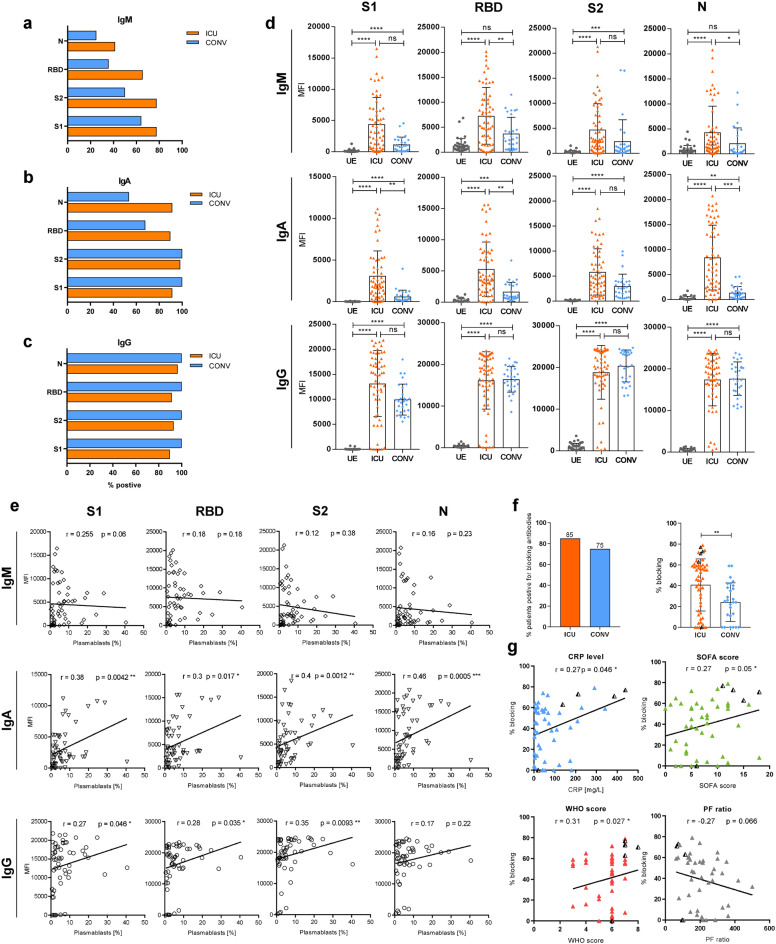
Plasmablasts contribute to antibody development and blocking activity is associated with disease severity. **a**–**e** Luminex-based multiplex assay was used to detect IgM, IgA, and IgG antibodies against S1-, RBD, S2-, or N-antigen of SARS-CoV-2 in patient sera. **a**–**c** Percentage of seroconverted ICU (*n* = 58) and CONV (*n* = 28) patients for SARS-CoV-2-specific IgM (**a**), IgA (**b**), and IgG (**c**) antibodies. The threshold for positive samples was calculated based on the mean fluorescent intensity (MFI) of antibodies from 36 UEs + 2× standard deviation. **d** Antibody levels from UE (*n* = 36), ICU (*n* = 58), and CONV (*n* = 28) are displayed as MFI. **e** Correlation analysis between S- and N-specific antibodies from ICU patients and plasmablasts proportions. **f** Percentage of ICU (*n* = 58) and CONV (*n* = 28) which developed blocking antibodies against SARS-CoV-2 RBD (left) and efficacy of blocking activity (right), which was assessed by competitive ELISA. Efficient blocking was expressed as the percent blocking at a 1:50 serum dilution relative to a UE serum control. **g** Correlation analysis between COVID-19 severity markers (CRP levels, SOFA-, WHO score, and PF ratio) and blocking efficiency from ICU patients. Black triangles represent last samples from deceased patients. Statistical analysis: multigroup comparisons were performed using ANOVA test with Turkey multiple comparison test or Kruskal–Wallis with test with Dunn’s multiple comparison test. Two-group comparison was performed using Mann–Whitney test; Spearman correlation. *P < 0.05, **P < 0.01, ***P < 0.001, ****P < 0.0001

Based on the massive plasmablast expansion in ICU, we hypothesized that their presence in circulation might be accompanied by the appearance of SARS-CoV-2-specific antibodies. A Luminex-based multiplex assay was used to detect IgM, IgA, and IgG specific for the S1, the receptor-binding domain (RBD), and the S2 regions of the spike protein (S) and the nucleocapsid (N) antigen of SARS-CoV-2 in plasma of ICU and CONV, while UE served as controls. In ICU, high IgG relative concentrations (MFI) were detected against all four SARS-CoV-2 antigens with the exception of two B-cell-depleted patients, while IgM and IgA displayed broad variations of 45–100% positive samples (Fig. 2a–c). In contrast, CONV showed decreased proportions of seroconversion and lower concentrations of IgM and IgA (Fig. 2a–d). Since the ICU samples were obtained during acute infection, they were collected earlier in the course of disease than CONV samples, which was reflected by the different IgM, IgA, and IgG kinetics and, thus, illustrated a time-dependent antibody-class switch from IgM to IgG and IgA (Supplementary Fig. S4a, b). The decline of IgM and IgA levels 20 days after symptom onset (DASO) in ICU was accompanied by increased IgG levels that remained high up to 200 days after symptom onset in CONV (Supplementary Fig. S4a, b). No major differences in IgM, IgA, and IgG responses were detected between the S- and N-antigens arguing for humoral responses against multiple SARS-CoV-2 antigens (Fig. 2d).

To unravel a potential relationship between plasmablast expansion and the occurrence of S- or N-specific antibodies, we correlated the proportions of plasmablasts with the corresponding relative antibody levels. While no correlation was found between plasmablasts and IgM antibodies, there was a weak positive correlation between plasmablasts and S- and N-specific IgA and IgG (Fig. 2e). Moreover, a competitive ELISA was performed to analyze whether antibodies in ICU were able to block in vitro binding of SARS-CoV-2 RBD (wildtype) to the ACE2 receptor. Interference was calculated as percent blocking in the presence of ICU plasma (1:50 dilution) relative to UE control plasma without SARS-CoV-2 antibodies. Interestingly, 85% of ICU and 75% CONV plasma samples contained interfering antibodies (Fig. 2f), but the blocking efficacy of ICU antibodies was significantly higher (median 40%) than CONV antibodies (median 24%) (Fig. 2f). Especially deceased patients exhibited high blocking activity (>60%) except for one B-cell-depleted patient. Moreover, we correlated antibody-blocking efficacy with disease severity makers: C-reactive protein (CRP) level as a marker for systemic inflammation, sepsis-related organ failure assessment (SOFA) score for the extent of systemic organ failure, World Health Organizations (WHO) eight-point scale for COVID-19 trial endpoints (https://www.who.int/publications/i/item/covid-19-therapeutic-trial-synopsis) and PaO_2_/FiO_2_ (PF) ratio representing the pulmonary function. CRP levels, SOFA- and WHO scores showed weak positive correlations with the antibody-blocking efficacy in ICU, whereas PF ratios were negatively correlated. Interestingly, deceased patients displayed remarkably high CRP levels, SOFA-, and WHO scores and low PF ratios (Fig. 2g).

These results illustrate that the majority of ICU developed rapid and sustained IgG antibody responses specific for SARS-CoV-2 S- and N-proteins and their blocking efficacy seemed to increase with disease severity suggesting that antibodies were insufficient to protect from developing severe COVID-19.

### Expansion of activated and terminally differentiated T and NK cells in COVID-19 ICU patients

**Fig. 3:**
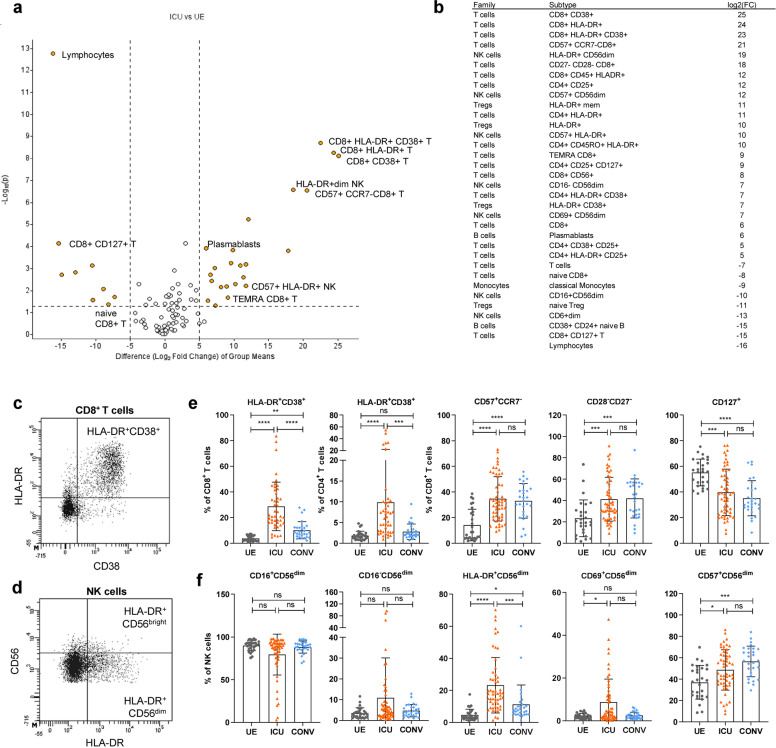
Expansion of activated and terminally differentiated T and NK cells in COVID-19 ICU patients. **a** Volcano plot visualizing two-group comparison of flow cytometry data including proportions of immune cells from ICU (*n* = 58) and UE (*n* = 29). **b** Significantly (*P* ≥ 0.05) altered immune cell subsets between ICU (*n* = 58) and UE (*n* = 29) ordered based on their fold change. **c**, **d** Representative flow cytometry plots. **e**, **f** Representative flow cytometry data of immune cell frequencies from UE (*n* = 29), ICU (*n* = 58) and CONV (*n* = 28). Statistical analysis: multigroup comparison was performed using ANOVA test with Turkey multiple comparison test or Kruskal–Wallis with test with Dunn’s multiple comparison test; *P < 0.05, **P < 0.01, ***P < 0.001, ****P < 0.0001

Next, we aimed to identify the major immune cell populations significantly altered in ICU versus UE to determine ICU-associated cellular patterns. Unbiased volcano plot analysis depicted a strong decline in lymphocytes but high frequencies of HLA-DR^+^CD38^+^ activated CD4^+^ and CD8^+^ T cells together with HLA-DR^+^ NK cells and CD57^+^ terminally differentiated T and NK cells in ICU compared to UE (Fig. 3a, b). These high frequencies of activated HLA-DR^+^CD38^+^ T cells (Fig. 3c) together with HLA-DR^+^ and CD69^+^ activated CD56^dim^ NK cells (Fig. 3d) were associated with acute COVID-19 and decreased with recovery in CONV (Fig. 3e, f). Proportions of CD28^−^CD27^−^ or CD57^+^CCR7^−^ terminally differentiated T and CD57^+^ NK cells were increased in ICU and remained elevated in CONV compared to UE (Fig. 3f). Consequently, CD127^+^CD8^+^ T-cell frequencies were decreased in ICU as well as in CONV compared to UE. Thus, severe SARS-CoV-2 infection is associated with strong activation and differentiation into late memory T and NK cells.

### In severe COVID-19, lymphocyte dynamics correlate with disease progression

**Fig. 4:**
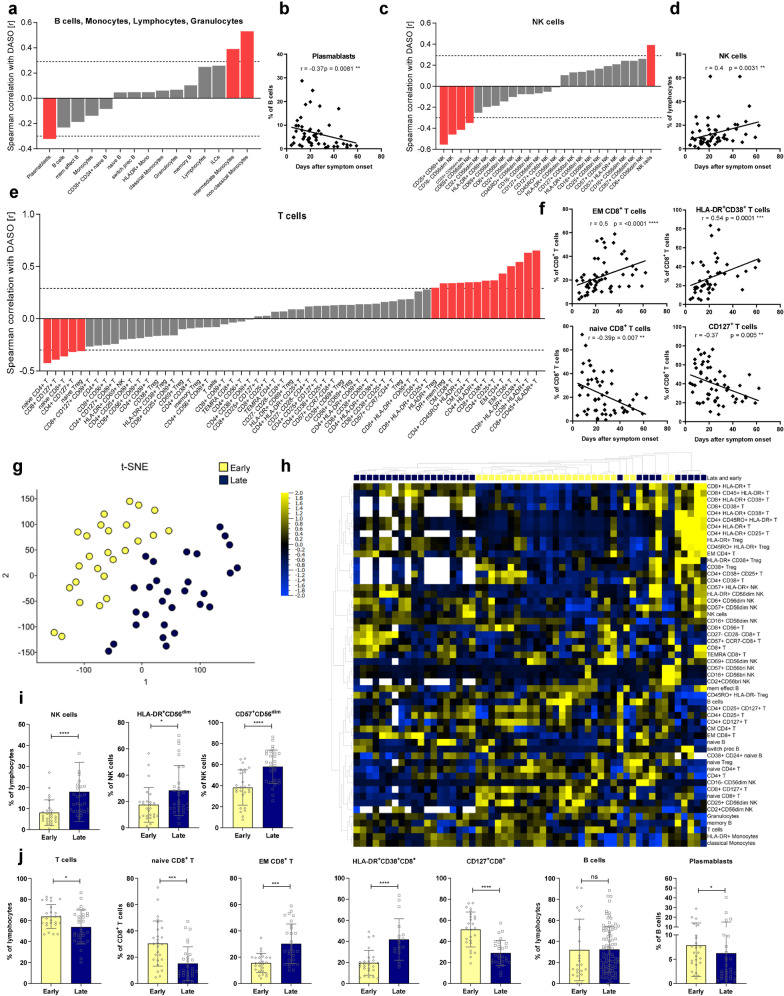
In severe COVID-19, lymphocyte dynamics correlate with disease progression. **a**, **c**, **e** Waterfall-plot representing correlation coefficient (*r*) of Spearman-correlation analysis between immune cell proportions of B cells, monocytes, lymphocytes, and granulocytes (**a**) or NK cells (**c**), or T cells (**e**) and disease duration as days after symptom onset (DASO) from ICUs (*n* = 58). Red columns represent significant results. Dotted lines represent the minimal Spearman-correlation coefficient (*r*) required for significant correlation. **b**, **d**, **f** Spearman-correlation analysis between proportions of plasmablasts (**b**), NK cells (**d**), or different T-cell populations (**f**) and DASO from ICUs (*n* = 58). **G** tSNE analysis of flow cytometry data from ICU (*n* = 58). A variance-value cutoff of 0.305 was used to identify significant differences within the ICU cohort. Patients were classified into two groups. Blue: late subgroup (*n* = 32), yellow: early subgroup (*n* = 26). **h** Heatmap of flow cytometry data including 98 immune cell populations from ICU (*n* = 58). A variance-value cutoff of 0.035 was used to identify significant differences within the ICU cohort. Samples and immune cell subsets were ordered according to hierarchical clustering. Blue to yellow scale represents the expression values normalized to mean=0, var=1. Missing values are displayed in white. **i**, **j** Representative immune cell proportions from early (*n* = 26) and late patients (*n* = 32) within the ICU cohort. Statistical analysis: Unpaired *t* test or Mann–Whitney test; Spearman correlation. *P < 0.05, **P < 0.01, ***P < 0.001, ****P < 0.0001

In order to examine the implications of the significant changes within the lymphocyte compartment in ICU, they were correlated to disease severity (SOFA-, WHO scores, CRP level) and progression (days after symptom onset, DASO). Several immune cell subsets significantly correlated with DASO, for example, plasmablasts with a negative correlation, showing that plasmablasts developed early during severe disease but decreased with prolonged ICU hospitalization (Fig. 4a, b). In the myeloid compartment, proportions of CD14^+^CD16^+^ intermediate and CD14^−^CD16^+^ non-classical monocytes showed a positive correlation to DASO. In parallel, NK cells increased with a simultaneous reduction of activated CD69^+^ or CD25^+^ NK cells over time (Fig. 4c, d). Moreover, different T-cell subsets correlated with disease duration, indicating a time-dependent T-cell differentiation caused by severe COVID-19 (Fig. 4e). While naive CD8^+^, CD4^+^ T, and CD25^hi^CD127^lo^ Treg cells decreased after symptom onset, EM as well as HLA-DR^+^CD38^+^ activated CD8^+^ T cells accumulated over time (Fig. 4e, f). As expected, frequencies of naive CD8^+^ T cells negatively and EM CD8^+^ T cells positively correlated with patient age in ICU (Supplementary Fig. S5a, b). However, since no correlation was observed between COVID-19 disease duration and patient age, we conclude that age had a minor impact on T-cell differentiation during severe COVID-19 (Supplementary Fig. S5c). Remarkably, CD4^+^ T cells and CD8^+^ TEMRA T cells did not follow the usual age correlation in ICU compared to UE (Supplementary Fig. S5a, b).

Unlike disease progression, severity markers (SOFA-, WHO-score, CRP level) correlated with only a few immune cell subsets, suggesting that disease severity had only a minor effect on the immune cell distribution (Supplementary Fig. S6a). As expected, especially activated immune cells like CD69^+^CD8^+^ T cells and CD69^+^CD56^bright^ NK cells positively correlated with CRP levels.

Furthermore, t-distributed stochastic neighbor embedding (tSNE) (Fig. 4g) and unsupervised cluster analyses (Fig. 4h) revealed two distinct subgroups of ICU samples, which separated based on their DASO into a “late” and “early” subgroup (Fig. 4h and Supplementary Fig. S6b, S6c). The “late” ICU subgroup consisted of samples obtained between day 7 to 68 (mean 34 days), whereas the “early” ICU subgroup consisted of samples from day 4 to 29 after symptom onset (mean 15 days). The two groups did not display significant differences regarding their SOFA- and WHO scores or PF ratios but the late subgroup exhibited decreased CRP levels compared to the early subgroup (Supplementary Fig. S6d). However, detailed analyses of the immune cell compositions illustrated that these two subgroups could additionally be distinguished by several immune cell proportions (Fig. 4h–j). Particularly expression of the activation markers HLA-DR, CD38, and CD25 and frequencies of CD45RO^+^ or CD57^+^ memory T and NK cells increased with disease duration in ICU, whereas consequently, proportions of naive T and NK cells declined over time (Fig. 4h–j).

These results show that the alterations within the lymphocyte compartment are strongly time-dependent during the individual ICU period but are less directly associated with disease severity.

### Systemic inflammation and endothelial injury in COVID-19 patients

**Fig. 5:**
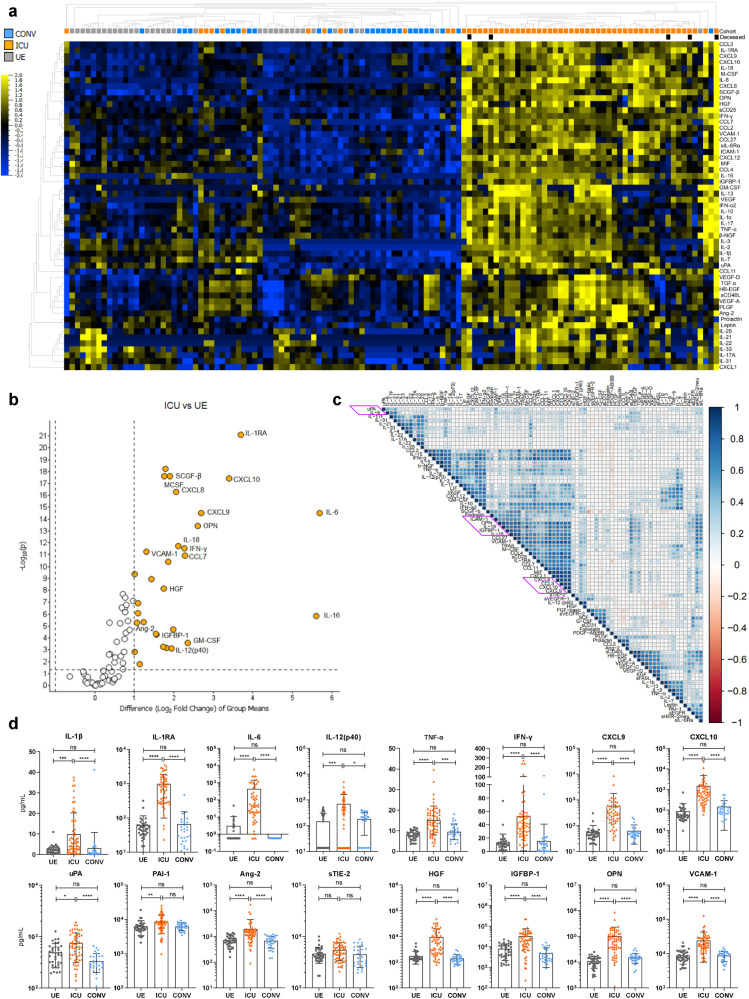
Systemic inflammation and endothelial injury in COVID-19 patients. Cytokine concentrations in patient sera were measured by Luminex-based multiplex assay. **a** Heatmap of cytokine data including 83 plasma proteins from UE (*n* = 36), ICU (*n* = 58), and CONV (*n* = 28). *P* value cutoff of 0.01 was used to identify significant differences among the three cohorts. Samples and immune cell subsets were ordered using to hierarchical clustering. Blue to yellow scale represents the expression values normalized to mean=0, var=1. Missing values are displayed in white. **b** Volcano plot visualizing a two-group comparison of plasma protein data from ICU (*n* = 58) and UE (*n* = 36). **c** Correlation matrix of 83 plasma proteins from ICUs. Spearman correlation was used to calculate correlation coefficients, which were displayed in circles. The strength of correlation was depicted by circle size. Cytokines were ordered using hierarchical clustering. Red to blue scale indicates the prevalence of each subset. **d** Representative cytokine concentrations from UE (*n* = 36), ICU (*n* = 58), and CONV (*n* = 28). Statistical analysis: Kruskal–Wallis test with Dunn’s multiple comparison test was performed. *P < 0.05, **P < 0.01, ***P < 0.001, ****P < 0.0001

With regards to the systemic microenvironment, we quantified concentrations of 83 plasma proteins in ICU, CONV, and UE including cytokines, chemokines, growth factors, and endothelial factors. Samples from ICU clearly clustered separately from CONV and UE and displayed significantly increased levels of 65 plasma proteins (Fig. 5a). Deceased ICU patients clustered in separated patterns, indicating different plasma protein signatures associated with fatal outcome. To identify the most statistically significant plasma proteins discriminating ICU from UE, we analyzed their levels based on the fold change between ICU and UE (Fig. 5b). The volcano plot illustrates a COVID-19-associated plasma protein profile composed of pro-inflammatory cytokines and chemokines (e.g., IL-6, CXCL10) in addition to growth factors (e.g., granulocyte–macrophage colony-stimulating factor (GM-CSF)) and endothelial factors (e.g., osteopontin (OPN), angiopoietin-2 (Ang-2)). In particular, chemokines and endothelial activation markers correlated with each other in clusters (Fig. 5c), indicating a co-regulated secretion and suggesting that inflammation and endothelial injury may collectively contribute to severe COVID-19. Furthermore, we observed high variability in the respective plasma proteins levels within the ICU cohort, as previously observed for immune cell populations (Fig. 5d and Supplementary Fig. S7). In addition to classical inflammation-associated cytokines (e.g., IL-1β, IL-6, TNF-α, IFN-γ) ICU displayed increased levels of soluble interleukin-6 receptor α (sIL-6Rα), urokinase-type-plasminogen activator (uPA), and its antagonist plasminogen-activator inhibitor 1 (PAI-1) (Fig. 5d and Supplementary Fig. S7). Whereas Ang-2, a central mediator of endothelial activation, was elevated, the concentrations of its soluble angiopoietin receptor 2 (sTIE-2) were stable in ICU, CONV, and UE, suggesting regulation at the ligand and not the receptor level. Endothelial activation in ICU was further detectable by significantly higher insulin-like growth factor-binding protein 1 (IGFBP-1), OPN, and vascular cell adhesion protein 1 (VCAM-1) levels, accompanied by several others. Organ contribution could be seen by higher hepatocyte growth factor (HGF) levels, for instance (Fig. 5d). Of note, the plasma protein profile of CONV displayed no significant alterations compared to UE.

In summary, we observed a highly inflammatory profile of plasma proteins in ICU, indicating strong immune system activation due to SARS-CoV-2 infection. Furthermore, high levels of endothelial factors suggest that endothelial injury may be a crucial contributor to severe COVID-19 progression.

### Core plasma protein signature of severe COVID-19 consists of eleven inflammatory mediators and endothelial factors

**Fig. 6:**
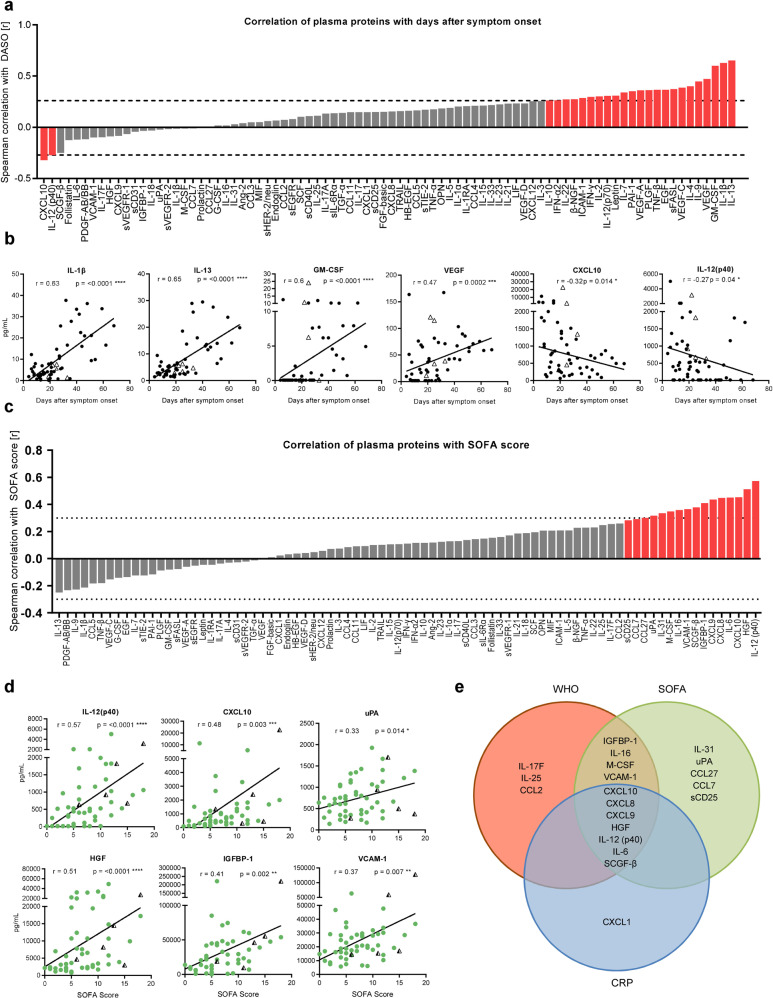
Core plasma protein signature of severe COVID-19 consists of 11 inflammatory mediators and endothelial factors. Cytokine concentrations in patient sera were measured by Luminex-based multiplex assay. **a**, **c** Waterfall-plot representing correlation coefficient (*r*) of Spearman-correlation analysis between cytokines and disease duration as DASO (**a**) or SOFA score (**c**) from ICUs (*n* = 58). Red columns represent significant results. Dotted lines represent the minimal Spearman-correlation coefficient (*r*) required for significant correlation. **b** Spearman-correlation analysis between representative cytokines and DASO. **d** Spearman correlation analysis of representative cytokines with SOFA score. **e** Venn diagram displaying significantly positive correlations between plasma proteins and CRP levels, SOFA-, and WHO score. Triangles represent the last samples from deceased patients. Statistical analysis: Spearman correlation. *P < 0.05, **P < 0.01, ***P < 0.001, ****P < 0.0001

Based on the extensive secretion of immunomodulating proteins and endothelial factors in ICU, we aimed to examine their implications for disease severity (SOFA-, WHO scores, and CRP levels) and disease duration (DASO). Several plasma protein levels positively correlated with disease duration such as IL-13, IL-1β, GM-CSF, and the vascular endothelial growth factor (VEGF) showing an extraordinary increase beyond day 20 after symptom onset (Fig. 6a, b). In contrast, CXCL10 and IL-12(p40) concentrations significantly decreased over time. Deceased ICU patients displayed particularly low IL-1β and IL-13 levels, while CXCL10 and IL-12(p40) plasma concentrations were increased (Fig. 6b). High SOFA scores correlated with high levels of chemokines like CXCL10 and endothelial factors like HGF, IGFBP-1, and uPA (Fig. 6c, d). Based on the highly inflammatory profiles of these ICU patients, it was not surprising that correlation analyses with CRP levels and WHO scores showed almost identical patterns (data not shown). Comparing plasma proteins positively correlated with either CRP levels, SOFA- or WHO scores, we detected an intersection of seven shared plasma proteins, representing the core signature associated with severe COVID-19 (Fig. 6e). This signature was composed of the pro-inflammatory cytokines IL-12(p40) and IL-6, the growth factor stem cell growth factor beta (SCGF-β), the chemokines CXCL8-10, and the endothelial factor HGF. Furthermore, IGFBP-1, IL-16, macrophage colony-stimulating factor (M-CSF), and VCAM-1 positively correlated with SOFA- and WHO scores, representing the contribution of the endothelium to severe COVID-19. Additional plasma proteins as IL-17F, IL-25, and CCL2 contributed to the unique WHO pattern, while IL-31, CCL27, CCL7, uPA, and sCD25 were included in the unique SOFA score-associated pattern and CXCL1 completed the CRP pattern.

Taken together, these signatures demonstrate that severe COVID-19 is characterized by extensive secretion of multifunctional interleukins, growth factors, and endothelial factors into the circulation, suggesting that SARS-CoV-2 causes more than a systemic inflammation but multiple endothelial injuries.

### Endothelial dysregulation as a major contributor to severe COVID-19

**Fig. 7:**
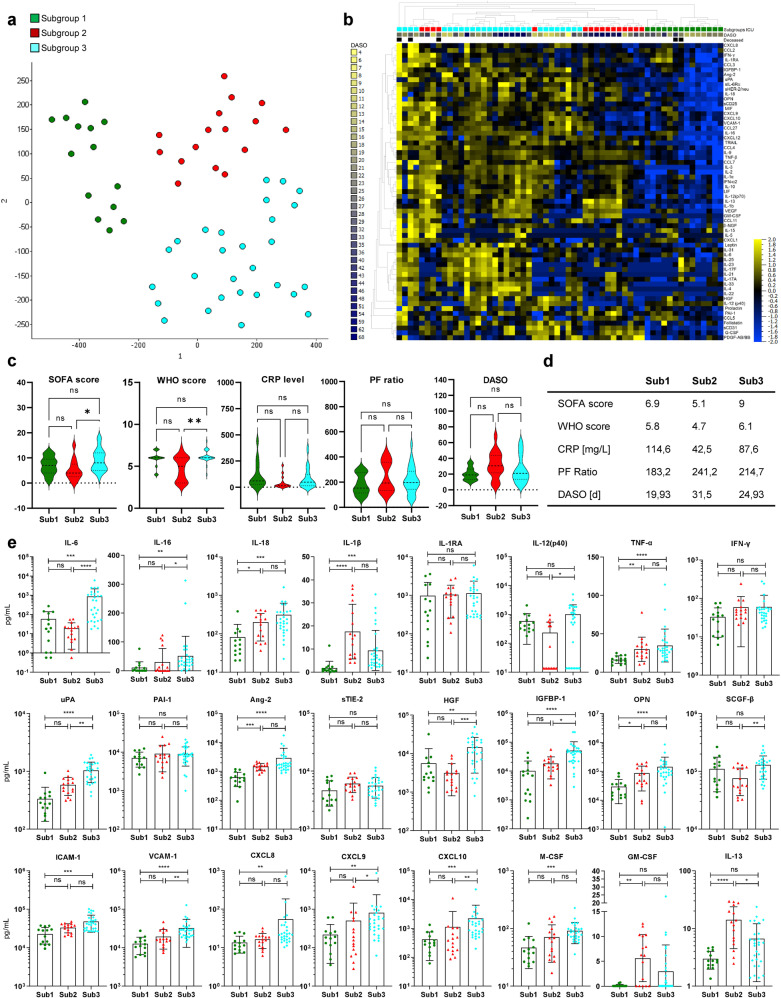
Endothelial dysregulation as a major contributor to severe COVID-19. Plasma protein concentrations in patient sera were measured by Luminex-based multiplex assay (**a**) tSNE analysis of 83 plasma protein data from ICU (*n* = 58). A variance-value cutoff of 0.146 was used to identify significant differences within the ICU cohort. Patients were classified into three subgroups. Green: subgroup 1 (sub1), red: subgroup 2 (Sub2), blue: subgroup 3 (Sub3). **b** Heatmap of 83 plasma protein from ICU (*n* = 58). A variance cutoff of 0.146 was used to identify significant differences among the three cohorts. Samples and cytokines were ordered using hierarchical clustering. Blue to yellow scale represents the expression values normalized to mean = 0, var = 1. Missing values are displayed in white. **c** Distribution of severity markers SOFA, WHO-score, CRP levels, PF ratio, and disease duration (DASO) among the three ICU subgroups. **d** Mean values of SOFA-, WHO-score, CRP level, PF ratio, and DASO for the three ICU subgroups. **e** Representative cytokine concentrations from ICU subgroups Sub1 (*n* = 14), Sub2 (*n* = 16), and Sub3 (*n* = 28). Statistical analysis: ANOVA test with Turkey multiple comparison test or Kruskal–Wallis with test with Dunn’s multiple comparison test were performed. *P < 0.05, **P < 0.01, ***P < 0.001, ****P < 0.0001

Finally, since we observed distinct plasma protein patterns in deceased ICU patients (Fig. 5a), we wanted to assess whether severe COVID-19 progression could be associated with distinct profiles of ICU patients. Unsupervised tSNE analysis of plasma proteins revealed three different subgroups within the ICU cohort (Fig. 7a) that clearly clustered separately, with the highest cytokine concentrations in subgroup 3 (Sub3) followed by subgroup 2 (Sub2) and subgroup 1 (Sub1) (Fig. 7b). Furthermore, Sub1-3 differed by their disease severity (SOFA-, WHO scores, CRP levels, PF ratio) and disease progression (DASO) (Fig. 7c, d). Of note, Sub2 contained ICU patients (*n* = 16) with the lowest CRP levels, SOFA- and WHO scores but highest PF ratios and with the longest disease durations (DASO) plus one deceased patient with chronic renal failure after renal transplantation (Fig. 7c, d). On the contrary, Sub3 (*n* = 28) displayed the highest SOFA- and WHO scores among the three subgroups containing two deceased patients. The lowest PF ratios and highest CRP levels were observed for Sub1 (*n* = 14) with two deceased patients. The plasma protein profiles demonstrated clear differences among these three ICU subgroups (Fig. 7e). In general, the lowest cytokine levels were observed for Sub1, which nevertheless still displayed significantly higher levels of key cytokines and chemokines like IL-6, IFN-γ, and CXCL10 compared to UE (Supplementary Fig. S8). Sub3 exhibited the strongest inflammatory signature with high levels of pro-inflammatory cytokines like IL-6, IL-18, and TNF-α in addition to sIL-6Rα and the chemokines CXCL8-10 (Fig. 7d and Supplementary Fig. S8). Remarkably, Sub3 had the highest levels of uPA, Ang-2, HGF, IGFBP-1, OPN, intercellular adhesion molecule 1 (ICAM-1), and VCAM-1 demonstrating endothelial activation, damage, and multiorgan failure (Fig. 7d and Supplementary Fig. S8). Most characteristics for Sub2 was an extensive secretion of IL-1β, IL-13, GM-CSF, and VEGF (Fig. 7d and Supplementary Fig. S8), which was already observed as “late” ICU signature (Fig. 6b). Compared to Sub3, Sub2 exhibited significantly lower IL-6 and IL-16 levels but concentrations of IL-18, TNF-α, Ang-2, and OPN were still elevated compared to Sub1 and UE (Fig. 7d and Supplementary Fig. S8). However, concentrations of tissue factors like IGFBP-1, uPA, and HGF were comparable to UE, suggesting less endothelial dysfunction in Sub2 (Supplementary Fig. S8).

In summary, the plasma protein profile of Sub3 implies massive inflammation, endothelial injury, and impairment of multiple organs, which is further supported by their high SOFA- and WHO scores. Patients from Sub2 had longer disease duration and decreased SOFA- and WHO scores compared to patients from Sub1 and Sub3. Together with decreased pro-inflammatory markers and endothelial factors, this indicates a gradual recovery from COVID-19 for Sub2 patients. Lastly, based on their plasma protein profile and clinical data, Sub1 patients are considered as patients at the beginning of a severe COVID-19 progression with massive inflammation but restricted endothelial damage and limited impairment of multiple organs.

## Discussion

Despite the intense global COVID-19 research, it is still incompletely understood which molecular mechanisms essentially contribute to severe disease progression and mortality. High-resolution single-cell-transcriptomic analyses focused almost exclusively on immune cells and proposed treatment-relevant signatures.^16^ Therefore, we aimed at highlighting the interface between the immune and endothelial systems in order to define signatures of severe COVID-19 with the potential to develop novel therapeutic strategies. In our study, we analyzed the cellular and humoral immune response in ICU and CONV, including a variety of plasma proteins as they serve as key mediators of inflammation and endothelial integrity.

In the peripheral blood of ICU, we identified major changes in the distribution of immune cell populations with a massive expansion of granulocytes along with a severe lymphopenia. Even though lymphopenia has been described as hallmark for severe COVID-19, it remains elusive whether it is caused by tissue infiltration or destruction of lymphocytes.^17^ As indicated by the T-cell signature of CONV, SARS-CoV-2 infection causes a differentiation of naive into effector CD8^+^ T cells, further supported by the significant increase of EM CD8^+^ T cells over time. Thus, the time-dependent differentiation of several immune cell populations could explain the high variability among proportions and numbers of immune cells in ICU. Several changes like the differentiation to memory T and NK cells persisted also in CONV arguing for long-term effects that may contribute to Long-COVID.

The immune signature of COVID-19 ICU displayed a highly activated phenotype, particularly of T and NK cells characterized by a HLA-DR^+^CD38^+^ and CD127^−^ phenotype. Up to 80% of CD8^+^ T cells in ICU were HLA-DR^+^CD38^+^, demonstrating strong T-cell activation in response to SARS-CoV-2. The appearance of HLA-DR^+^CD38^+^CD8^+^ T cells has also been described for other viruses like HIV and Influenza A and was associated with worse outcomes and impaired T-cell function.^18,19^ For COVID-19 others have shown that conversely to their activated phenotype HLA-DR^+^CD38^+^CD8^+^ T cells also expressed exhaustion markers like PD-1 but were still functional by producing e.g., IFN-γ, granzyme B, and perforin, suggesting that these cells are not generally functionally impaired by their PD-1 expression.^20,21^

Regarding the humoral immunity to SARS-CoV-2, several studies demonstrated an extraordinary B-cell response with the absence of germline centers in deceased COVID-19 patients,^22^ an extensive class switching to IgG and IgA accompanied by limited somatic hypermutation,^23^ as well as TGF-β instructed SARS-CoV-2 unspecific B-cell responses.^24^ As others have previously shown,^8^ we observed a significant expansion of plasmablasts in severe COVID-19 patients accompanied by IgA and IgG SARS-CoV-2-specific antibodies. In our study, ICU not only produced high levels of S- and N-specific antibodies but the majority of these antibodies also blocked the binding of SARS-CoV-2 RBD to ACE2 in vitro, indicating the development of functional antibodies. Nevertheless, these patients developed severe COVID-19 symptoms despite the presence of blocking antibodies, suggesting that the humoral immune response alone may be insufficient to prevent critical disease progressions. As the development of SARS-CoV-2-specific antibodies in ICU together with the activation and differentiation of T and NK cells proves that these patients are immunocompetent, other mechanisms are likely to be involved in severe COVID-19 progression.

In our study, we observed that severely ill COVID-19 patients were characterized by a massive release of several plasma proteins indicating endothelial damage and a previously reported COVID-19-associated cytokine storm.^25^ We identified seven core plasma proteins i.e., CXCL8-10, HGF, IL-6, IL-12(p40), and SCGF-β for severe COVID-19 that were associated with organ failure (SOFA score), inflammation (CRP level), and lung dysfunction (WHO-score). Furthermore, elevated levels of IGFBP-1, M-CSF, VCAM-1, and IL-16 correlated with high SOFA- and WHO scores, indicating a connection of these soluble mediators to multiple organ and endothelial injuries. Overall, markedly elevated levels of these plasma proteins depict a highly inflammatory environment as well as endothelial activation and disruption and therefore are possible predictors for disease progression and outcome. This assumption is supported by the finding of Deng et al., who observed increased concentrations of CXCL8, SCGF-β, and HGF in severe compared to non-severe SARS-CoV-2-infected patients.^26^ In particular, they found HGF discriminating severe vs. non-severe COVID-19 with 84% sensitivity and 98% specificity. Moreover, elevated CXCL9 concentrations could distinguish SARS-CoV-2-associated multisystem inflammatory syndrome in children from other hyperinflammatory syndromes like Kawasaki disease and macrophage activation syndrome, underlining the potential of CXCL9 as a predictive marker in COVID-19 disease progression.^27^

CXCL8-10 as well as IL-6 and IL-12(p40) are highly pro-inflammatory mediators and elevated in cytokine storm release syndrome and systemic diseases.^25,28^ IL-6 serves as a major orchestrator for systemic inflammation through induction of the hepatic acute phase response. Furthermore, high circulating levels of IL-6 as in a COVID-19-associated cytokine storm result in *trans-*signaling allowing IL-6 together with sIL-6Rα to activate cells not expressing the membrane-bound IL-6R, like endothelial cells.^25^ As a result of the IL-6-mediated activation, E-cadherin expression is reduced on endothelial cells leading to vascular hyperpermeability. Moreover, not only IL-6 but also HGF has been reported to directly contribute to endothelial disruption and vascular leakage.^29^ IGFBP-1 and its ligand IGF-1 are known to be elevated in severe lung injuries like idiopathic pulmonary fibrosis and ARDS.^30,31^ The fact that elevated IGFBP-1 levels are associated with hypoxia further suggests that IGFBP-1 may be a predictor of lung dysfunction and tissue damage.^32^ Since soluble VCAM-1 and M-CSF are additional indicators of endothelial activation and were identified as hallmark plasma proteins for severe COVID-19,^33,34^ we propose that endothelial activation and disruption are major drivers of severe COVID-19, which has not been demonstrated in parallel to immune dysregulation before.

Endothelial damage together with elevated levels of pro-inflammatory cytokines or endothelial factors are not exclusive for COVID-19 but described for other severe virus infections as well. Dengue virus infection is associated with vascular leakage^35^ in addition to elevated levels of HGF and Ang-2, which increased with disease severity in children suffering from dengue hemorrhagic fever^36,37^. Decreased barrier function and multiorgan failure^38^ are characteristic for patients with Ebola infection together with increased levels of the pro-inflammatory cytokine and chemokines IL-6 and CXCL8-10.^39^ Furthermore, enhanced secretion of uPA and PAI-1 were found in Ebola-infected non-human primates at an early stage of infection, indicating an imbalanced coagulation system, as it has been observed for COVID-19 as well. PAI-1 concentration was also increased in influenza-infected mice.^40^ In humans, severe influenza infection is, like COVID-19, associated with ARDS and elevated levels of pro-inflammatory cytokines, chemokines, and endothelial factors like IL-6, TNFα, CXCL8, CXCL10, and HGF.^41,42^ These results argue for a crucial role of the endothelial integrity in several severe virus infections and, thus, indicate that endothelial cells could serve as a therapeutic target. In line with this, Xu et al. have recently shown that expression of KLF2, a master transcriptional regulator of endothelial integrity and homeostasis is downregulated in vitro in HMVEC-L endothelial cells after treatment with the serum of severe COVID-19 patients. Interestingly, atorvastatin treatment reversed these effects in vitro, suggesting statins as a therapeutic option to control endothelial damage and to retain vascular homeostasis in severe COVID-19.^43^

The different plasma protein profiles from ICU suggest a crucial role of endothelial damage in severe COVID-19 and link the inflammatory response to endothelial barrier disruption. Focusing on ICU, unsupervised cluster analyses identified three subgroups based on their plasma protein profiles and implied a development from a primarily inflammatory phenotype in Sub1 to additional endothelial activation and disruption in Sub3 to endothelial reconstitution and decline of inflammation in Sub2. A highly inflammatory phenotype with elevated plasma levels of IL-6, IL-12(p40), IFN-γ, and CXCL10 characterized Sub1 patients without signs of endothelial damage. In contrast, remarkably high levels of HGF, Ang-2, OPN, and VCAM-1 in Sub3 represented extended endothelial activation and disruption in addition to inflammation also mediated by IL-6, CXCL10, IL-18, and IL-12(p40). Furthermore, elevated secretion of key regulators of fibrinolysis like uPA and PAI-1 were unique features of Sub3 and illustrated a dysregulation of fibrinolysis and coagulation in addition to endothelial damage and inflammation. Since microthrombotic patterns are well described for severe COVID-19^44^ and represent a serious complication, these results support the assumption of Sub3 as high-risk patients with systemic inflammation, tissue damage, and impaired coagulation and therefore poor survival prospects.

Sub2 was characterized by lower levels of pro-inflammatory cytokines and endothelial factors like IL-6, IL-12(p40), uPA, HGF, and IGFBP-1, together with decreased SOFA- and WHO scores suggesting a decline in inflammation and endothelial damage. Furthermore, Sub2 exhibited the highest levels of the anti-inflammatory cytokines IL-13 and IL-1β, which correlated with decreased SOFA- and WHO scores, as well as CRP levels. While IL-13 was reported to be required for recovery after acute lung injury by regulating local levels of IL-6, CCL2/MCP-1, and G-CSF, IL-1β is usually known as a pro-inflammatory cytokine related to ARDS.^45,46^ However, other studies propose that IL-1β is involved in tissue healing and has beneficial effects on endothelial repair after ARDS.^47^ In addition, GM-CSF and VEGF are further factors elevated in Sub2 and significantly increased over time together with IL-1β and IL-13. Correspondingly, GM-CSF and VEGF also have been described to essentially contribute to tissue repair and wound healing.^48,49^ These observations indicate that elevated levels of IL-1β, IL-13, GM-CSF, and VEGF after 20 days of symptom onset are predictors for tissue repair and improved health condition of severe COVID-19 patients. The observation that Long-COVID syndrome is characterized by an impairment of several organ systems including pulmonary, cardiovascular, and neurophysiological symptoms^14^ further suggests a continuous contribution of the endothelial-epithelial barrier to Long-COVID. Therefore, knowing the mechanisms involved in the establishment and recovery of COVID-19, focusing especially on the endothelium, may be essential for more precise diagnostics for Long-COVID and even treatment strategies.

In conclusion, we propose that in severe cases SARS-CoV-2 infection leads to a massive local inflammation developing into a dysregulated immune activation with cytokine and chemokine release. The combination of this inflammation with a systemic endothelial activation can proceed towards capillary leakage and multiorgan failure. To the best of our knowledge, the present study shows for the first time that endothelial damage is another major driver of COVID-19 severity together with substantial immune dysregulation. Thus, severe and life-threatening conditions of COVID-19 patients are not only characterized by a highly activated immune phenotype and pro-inflammatory cascades but also by substantial endothelial injuries, which may explain multiorgan involvement in severe COVID-19.

### Limitations of the study

The limitations of our observational study include the single-center setting with a rather small and heterogeneous patient cohort. Furthermore, samples of ICU patients were obtained at variable time points during disease according to the clinical procedure in Lower Saxony with MHH serving as a tertiary care center for severe cases in need of advances life support. Therefore, a prospective study design with defined time points was not feasible. Nevertheless, time-dependent alterations could be identified by combining patient data, a procedure that has been utilized for many COVID-19 studies. Of course, further studies are needed to define the therapeutic consequence of our observations, especially with respect to their impact on long-COVID.

## Materials and methods

### Study design

In total 25 patients from the intensive care unit (ICU), and 17 convalescent patients were recruited to this observational study between April and September 2020 at Hannover Medical School (MHH). The convalescent cohort included former ICU patients. ICU and CONV had confirmed SARS-CoV-2 infection with the D614G virus-variant via viral PCR. For some patients, samples were taken at multiple time points leading to a sample size of 58 for ICU patients and 28 for convalescent patients. Five patients from ICU died and were visualized with the last sample obtained before decease. The clinical severity of COVID-19 was assessed on different parameters and the time point of disease severity assessment was consistent with that of blood collection. World Health Organizations (WHO) eight-point score for COVID-19 trail endpoints was used to classify the severity of COVID-19 (https://www.who.int/publications/i/item/covid-19-therapeutic-trial-synopsis) and is based on the requirement of non-invasive oxygen supply and mechanical ventilation or extracorporeal membrane oxygenation. C-reactive protein (CRP) level was used as inflammation marker. The sepsis-related organ failure assessment (SOFA) score determines organ function and the extent of systemic organ failure in patients at ICU. This score is based on six organ systems, which are the respiratory system, nervous system, cardiovascular system, liver, kidney, and coagulation. PF ratio represented the individual pulmonary function. Clinical and demographical characteristics of study participants are summarized in Supplementary Table 1. Of the 25 ICU patients, 12 received remdesivir, and 6 received Tocilizumab treatment. In total, 29 unexposed individuals (UE) were enrolled as the control group, some also at several time points leading to a sample size of 36 for UE.

### Quantification of cells from EDTA blood via TruCount™ analysis

Absolute cell numbers were calculated from whole blood using BD Trucount™ Tubes (BD Biosciences), following the manufacturer’s instructions.

### Flow cytometry

Flow cytometry analyses were performed as recommended by the guidelines of leading European scientists of immunology and flow cytometry communities.^50^ Whole blood EDTA samples (100 µl) were incubated with antibodies for surface staining in FACS Buffer (0,1% NaN_3_, 2,5% FCS in PBS) at 4 °C for 30 min and followed by 15 min erythrocyte lysis using 1× BD Lysing Solution. Cells were washed with PBS prior to acquisition. All antibodies used for flow cytometry analyses are listed in Supplementary Table 2. Cells were acquired and analyzed on a LSRII flow cytometer (BD Biosciences, USA) using FACS Diva software (v8.0).

### Multiplex assays

Luminex-based multiplex assays were used to quantify plasma proteins and SARS-CoV-2 S- and N-specific antibodies.

Plasma proteins were measured using the Bio-Plex Pro^TM^ Human Assays (Bio-Rad, Hercules, USA): cytokine screening panel plus ICAM-1 and VCAM-1 (12007283, 171B6009M, 171B6022M), Cancer Biomarker Panel 1, and Panel 2 (171AC500M, 171AC600M) and Th17 cytokine Panel 15-Plex (171AA001M) following the manufacturer’s instructions with thawed plasma and which was diluted twofold with assay buffer. Standards were reconstituted and prepared as described in the manufacturer’s instructions. Standard curves and concentrations were calculated using the Bio-Plex Manager 6.1 software.

SARS-CoV-2 S- and N-specific antibodies were detected using the SARS-CoV-2 Antigen Panel 1 IgG, IgM, IgA assay (Millipore HC19SERM1-85K-04, HC19SERA1-85K-04, HC19SERG1-85K-04) following the manufacturer’s instructions. The assays were performed with thawed plasma, which was diluted 1:200 for ICU and CONV and 1:100 for UE with assay buffer.

The semi-quantitative readout is given as median fluorescence intensity (MFI) of >50 beads for each antigen and sample, acquired by the Bio-Plex 200 machine and the Bio-Plex Manager^TM^ Version 6.0 software (Bio-Rad Hercules, USA).

### Competitive ELISA

Competitive ELISA was performed to detect blocking antibodies against SARS-CoV-2 RBD. Plates were coated overnight at 4 °C with 50 µl/well of recombinant RBD (400 ng/ml). After three times washing, plates were blocked for 1–2 h at room temperature (RT) with PBS + 2% FCS. In total, 0.25 µg/ml biotinylated human ACE2 (Acro Biosystems) were added followed by 50 µl patient plasma (1:50 dilution). Plates were incubated at RT for 1–2 h and washed three times afterward. HRP-bound streptavidin (Merck-Millipore) was added and incubated at RT for 1–2 h, then washed 3–4 times. TMB substrate (BD Biosciences) was added and the reaction was stopped with 50 µl H_2_SO_4_ (0.5 M). Optical densities were measured at 450 nm. Efficient blocking was expressed as the percent neutralization at a 1:50 plasma dilution relative to a UE control serum.

### Statistical analyses

Statistical analyses of the data were performed with GraphPad Prism v7.0/v9.0 software (GraphPad Software). D’Agostino-Pearson omnibus normality test was calculated to assess data distribution. Parametric tests were performed where data were normally distributed, otherwise non-parametric tests were used. The statistical test used in each analysis is indicated in the figure legends. Correlation analyses were performed using Spearman rank-order correlation. Results were considered significant if *P* < 0.05.

Qlucore Omics Explorer (version 3.6, Qlucore) was used to generate principal component analysis (PCA) plots, heatmaps, tSNE plots, and volcano plots. Therefore data were normalized to mean=0 and var=1. Furthermore, cytokine/chemokine data were log-transformed for Qlucore Omics Explorer analyses. For each analysis, *P* value or variance used as a cutoff are indicated in the figure legends.

For the cytokine correlation matrix, Spearman’s rank-order correlation coefficient was visualized using the corrplot R package.

### Study approval

The study was approved by the Hannover Medical School Ethics Committee. All patients or participants provided written informed consent before participation in the study (9001 BO K, 968-2011).

## Supplementary information

Supplementary Figures

## Data Availability

The datasets used and/or analyzed to support the findings of this study are available in this paper or the Supplementary Information. Any other raw data that support the findings of this study are available from the corresponding author upon reasonable request.
