# Supplementary material for: Endothelial dysfunction contributes to severe COVID-19 in combination with dysregulated lymphocyte responses and cytokine networks
Source: Signal Transduct Target Ther. 2021 Dec 10;6:418. doi: 10.1038/s41392-021-00819-6 (PMC8661333; doi:10.1038/s41392-021-00819-6)
Supplement: Supplementary file 1 — Supplementary Figures [file 41392_2021_819_MOESM1_ESM.pdf]

Supplementary Materials for

Endothelial dysfunction contributes to severe COVID-19 in combination with dysregulated lymphocyte responses and cytokine networks

Authors: Louisa Ruhl[1], Isabell Pink[2], Jenny F. Kühne[1], Kerstin Beushausen[1], Jana Keil[1], Stella Christoph[1], Andrea Sauer[2], Lennart Boblitz[2], Julius Schmidt[2], Sascha David[3], Hans-Martin Jäck[4], Edith Roth[4], Markus Cornberg[5,6], Thomas F. Schulz[7,9,10], Tobias Welte[2,8], Marius M. Höper[2,8], Christine S. Falk[1,8,9*]

Correspondence to: falk.christine@mh-hannover.de

This PDF file includes:

Figures. S1 to S8
Tables S1 to S2

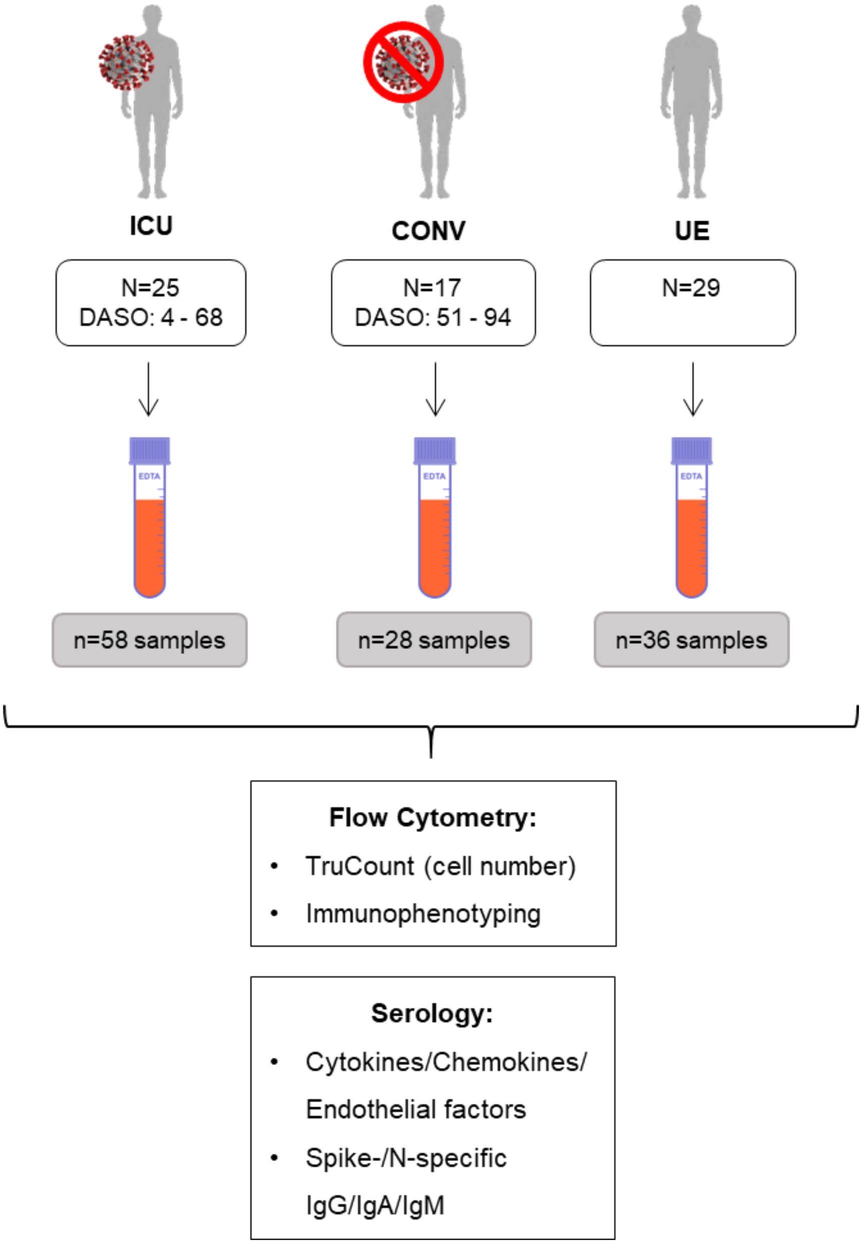

Fig. S1: Overview of the study design
UE: Unexposed donors, ICU: Intensive care unit patients, CONV: convalescent patients, DASO: Days after symptom onset

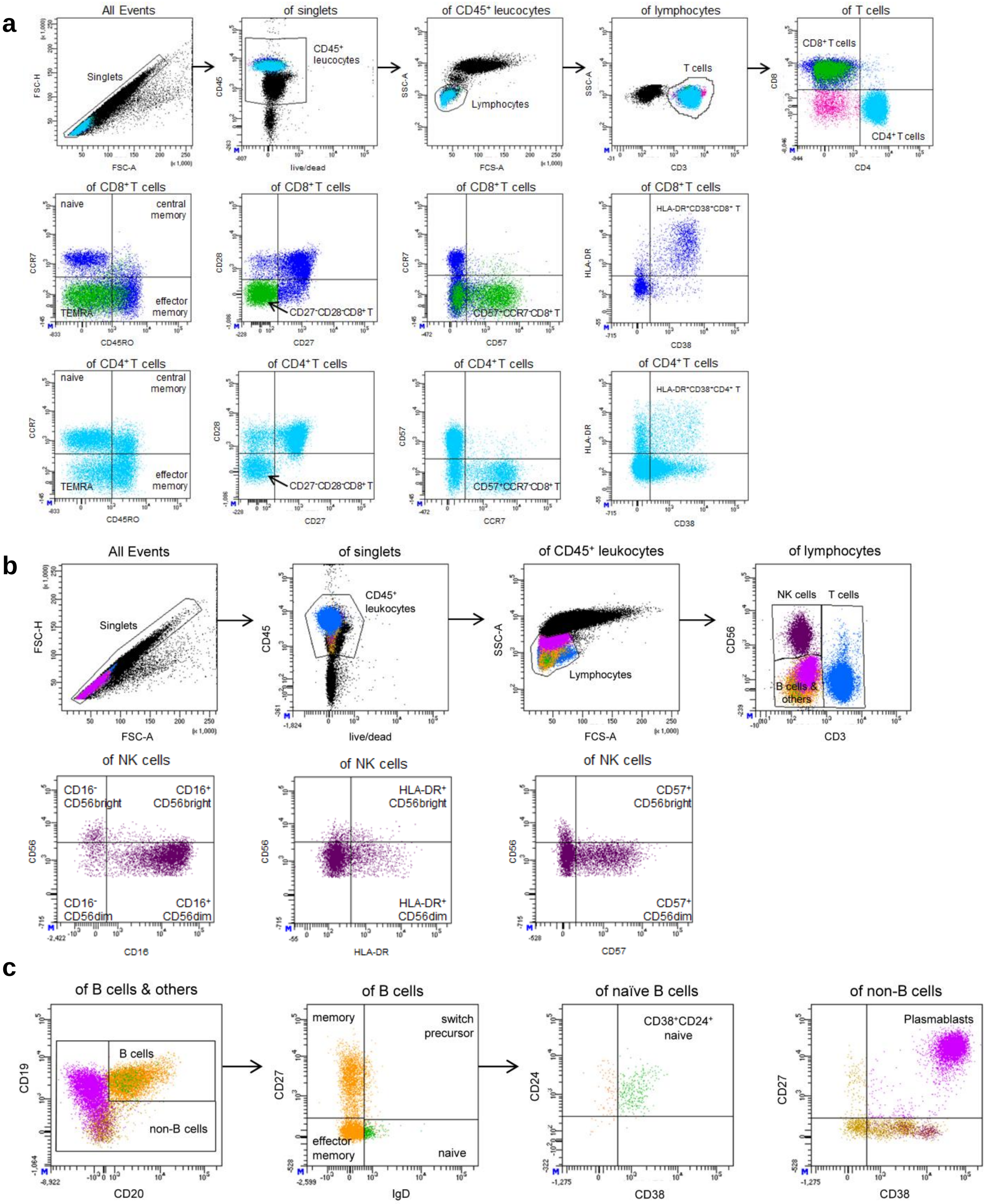

Fig. S2: Flow cytometry gating strategy.
Representative flow cytometry plots visualizing the gating strategy for **(a)** T cell subsets, **(b)** NK cell subsets and **(c)** B cell subsets.

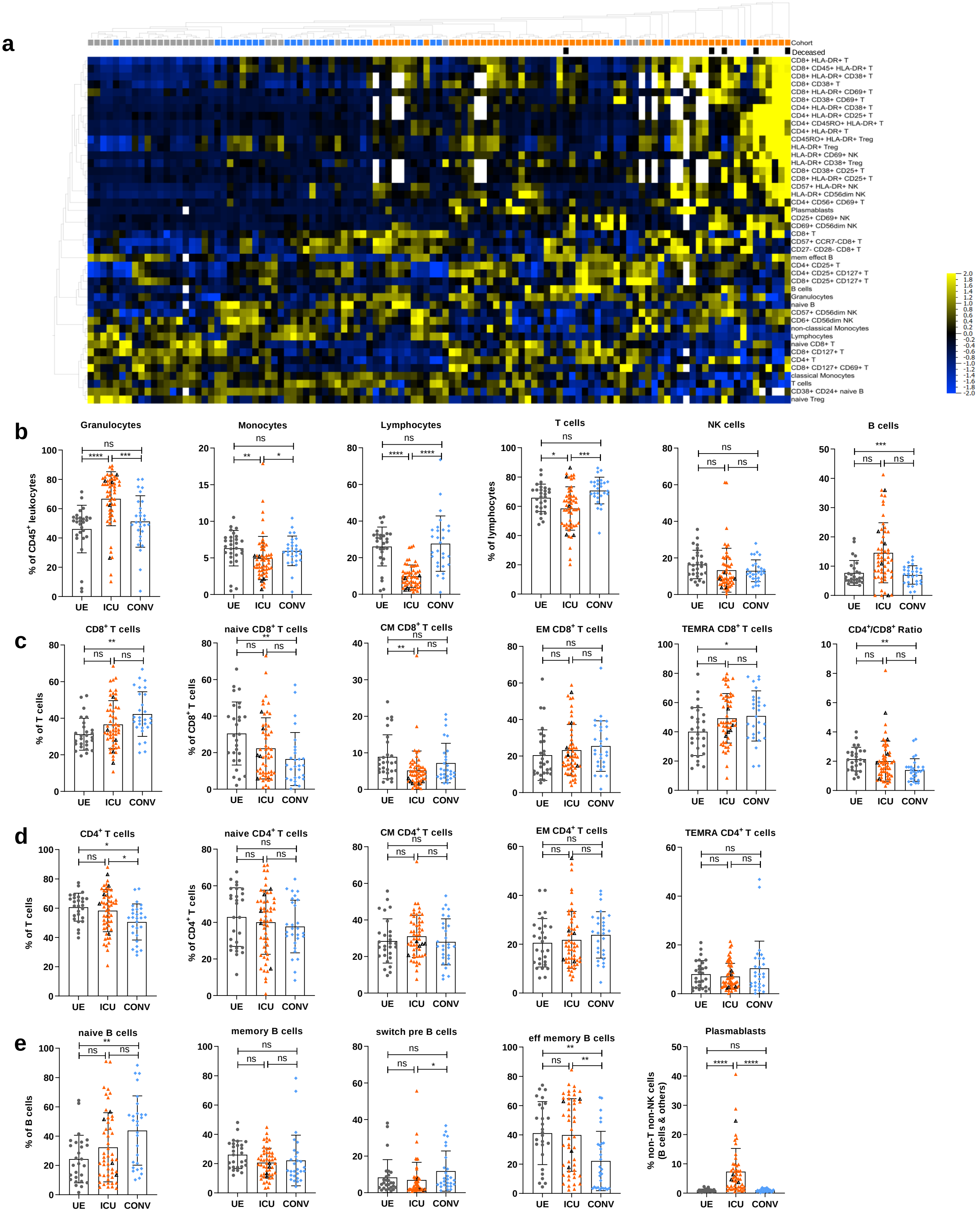

Fig. S3: Altered frequencies of certain immune cells in severe COVID-19 patients.

Immune cell distribution reflected by frequencies in patient blood was analyzed using flow cytometry. **(a)** Heatmap analysis. Multigroup comparison with a p-value cut off of 0.041 was used to identify significant differences among UE, ICU and CONV. **(b-e)** Frequencies of different immune cells in patient blood. T cells: naïve (CCR7+CD45RO-), central memory (CM, CCR7+CD45RO+), effector memory (EM, CCR7-CD45RO+) and TEMRA (CCR7-CD45RO-); B cells: naïve (IgD+CD27-) memory (mem, CD27+IgD-), switch precursor (switch pre, CD27+IgD+), effector memory (eff mem, IgD-CD27-) and plasmablasts (CD19+CD20-CD27+CD38+)

Black triangles represent last samples from deceased patients.

UE: Unexposed donors, ICU: Intensive care unit patients, CONV: convalescent patients, CM: central memory, EM: effector memory, mem eff: memory effector

Statistical analysis: ANOVA test with Turkey multiple comparison test or Kruskal-Wallis with test with Dunn's multiple comparison test were performed. *p < 0.05, **p<0.01, ***p<0.001, ****p<0.0001.

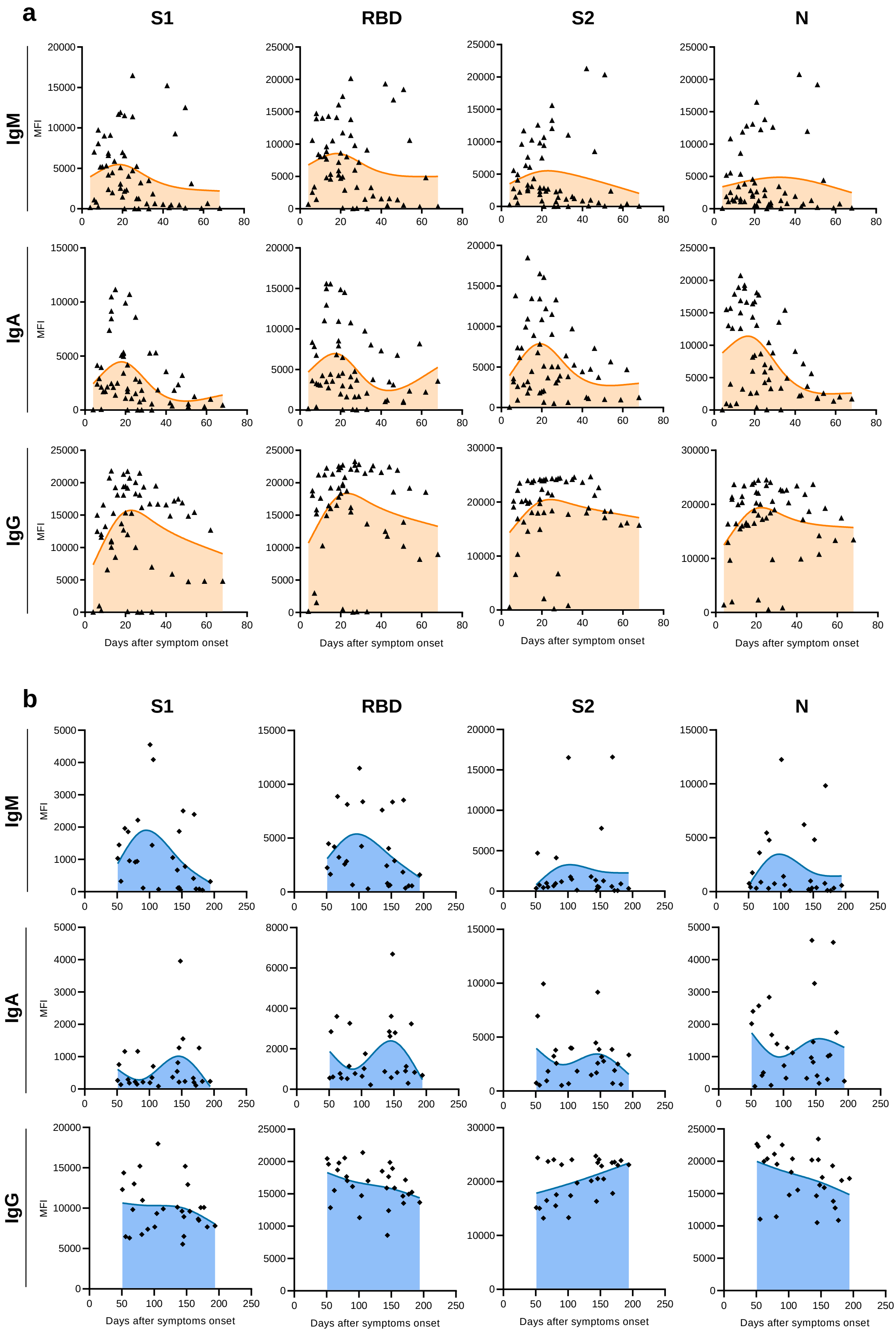

Fig. S4: Changes in SARS-CoV-2 specific antibody levels over time in ICU and CONV.
Luminex-based multiplex assay was used to detect SARS-CoV-2 specific IgM, IgA and IgG antibodies against S1-, S2-, N- or RBD-antigen of SARS-CoV-2 in patient sera. Antibody levels were plotted against days after symptom onset for **(a)** ICU and **(b)** CONV.
Curves were generated with LOWESS regression.

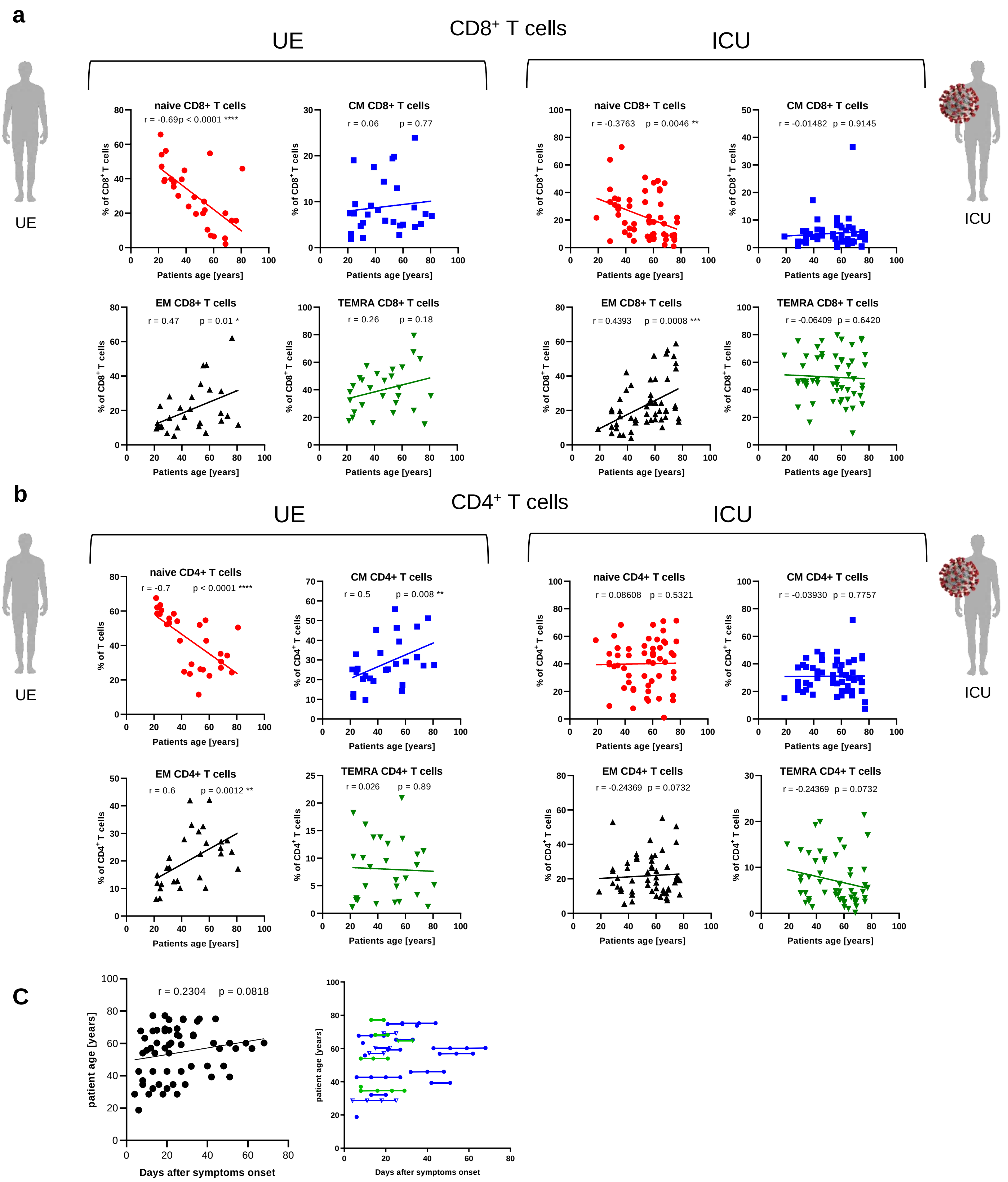

Fig. S5: Age-related changes in T cell subsets in ICU patients.
(a, b) Frequencies of CD8+ and CD4+T cell subsets in ICU patient blood, including naïve (CCR7+CD45RO-), central memory (CM, CCR7+CD45RO+), effector memory (EM, CCR7-CD45RO+) and TEMRA (CCR7-CD45RO-), were analyzed using flow cytometry. Frequencies were correlated to patient age. **(c)** Correlation between patient age and days after symptom onset (left). ICU patient age against days after symptom onset. Repeated samples from the same individuum are linked. Green dots represent samples from female patients. Blue dots represent samples from male patients. Triangles represent samples from deceased patients.
Statistical analysis: Spearman-Correlation. *p < 0.05, **p<0.01, ***p<0.001, ****p<0.0001.

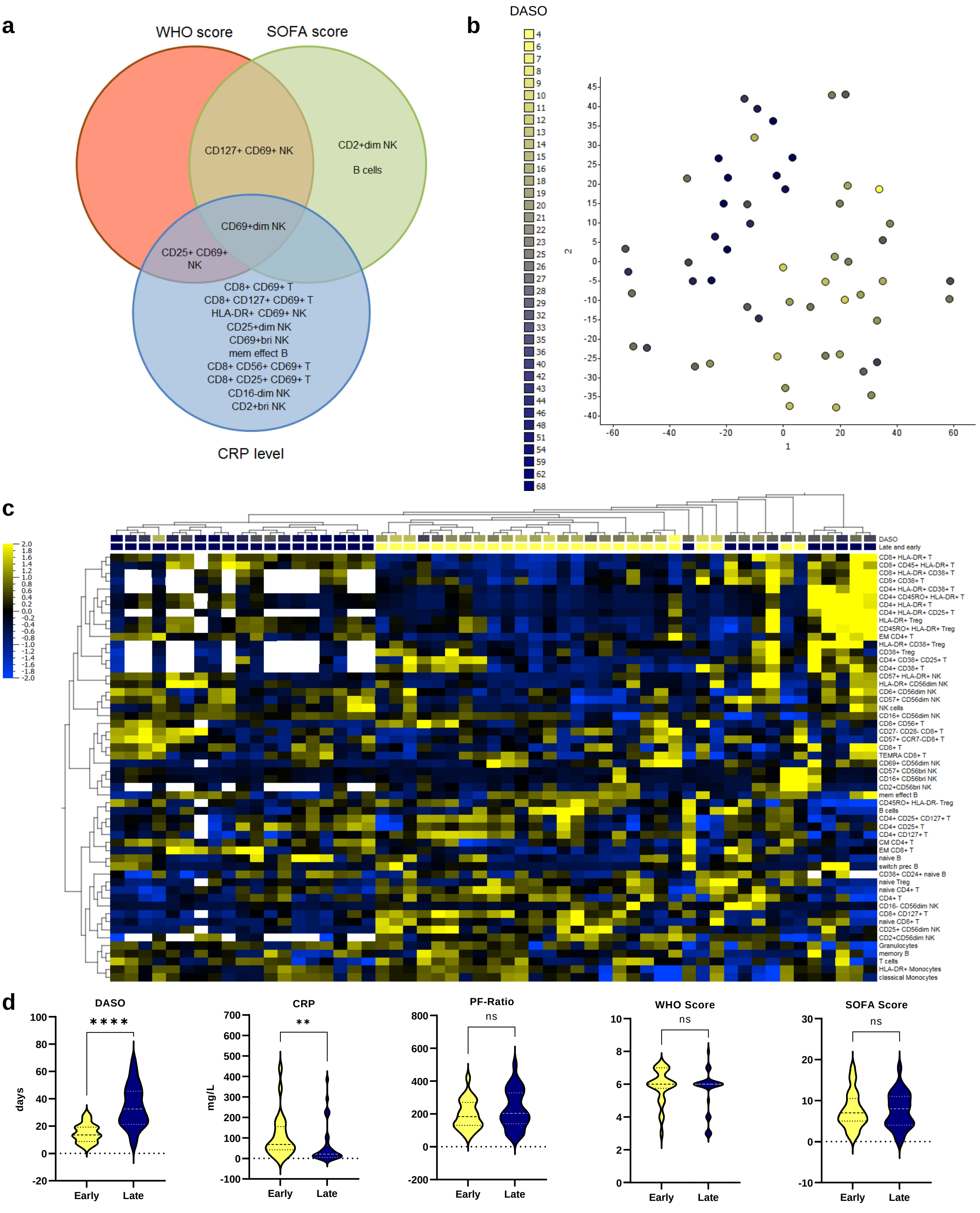

Fig. S6: Correlation between the immune cell composition of COVID-19 ICU patients and disease progression
(a) Venn-diagram displaying significantly positive correlations between immune cell subsets and CRP levels, SOFA- and WHO-score **(b)** tSNE analysis of flow cytometry data from ICUs. Variance-value cut off of 0.305 was used to identify significant differences within the ICU cohort. Patients samples were colored based on their days after symptom onset. **(c)** Heatmap of flow cytometry data including 98 immune cell populations from ICUs. Variance-value cut off of 0.035 was used to identify significant differences within the ICU cohort. Samples and immune cell subsets were ordered according to hierarchical clustering. Patients were classified into two groups. Blue: Late subgroup, yellow: early subgroup. Blue to yellow scale indicates the prevalence of each subset. Missing values are displayed in white. **(d)** Clinical parameters from early and late ICU patients.
DASO: Days after symptom onset
Statistical analysis: Unpaired t-test or Mann-Whitney test. *p < 0.05, **p<0.01, ***p<0.001, ****p<0.0001.

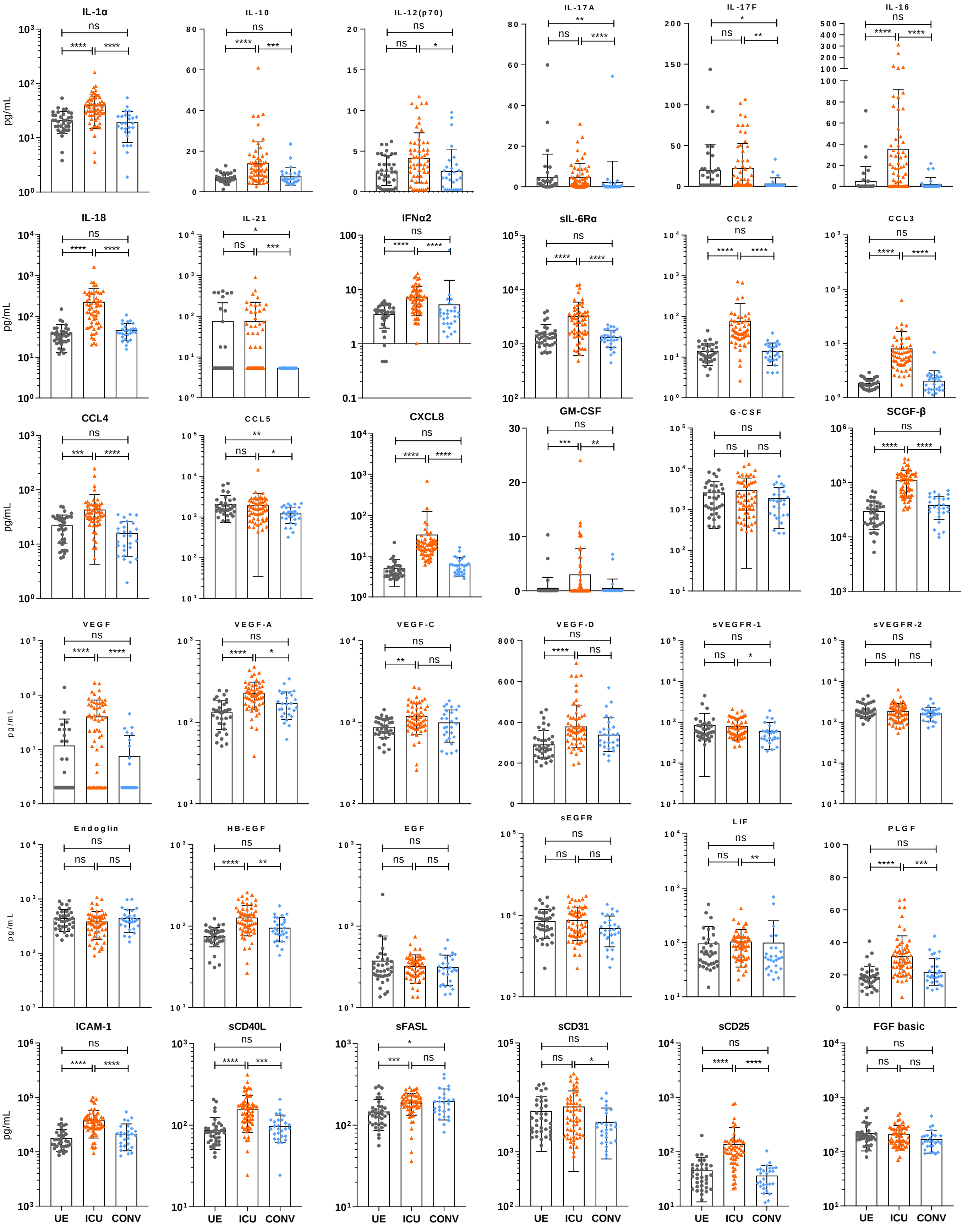

Fig. S7: Levels of cytokines, chemokines and endothelial factors in plasma of UE, ICU and CONV.
Luminex-based multiplex assay was used to analyze levels of cytokines and chemokines in plasma from UE, ICU and CONV patients.

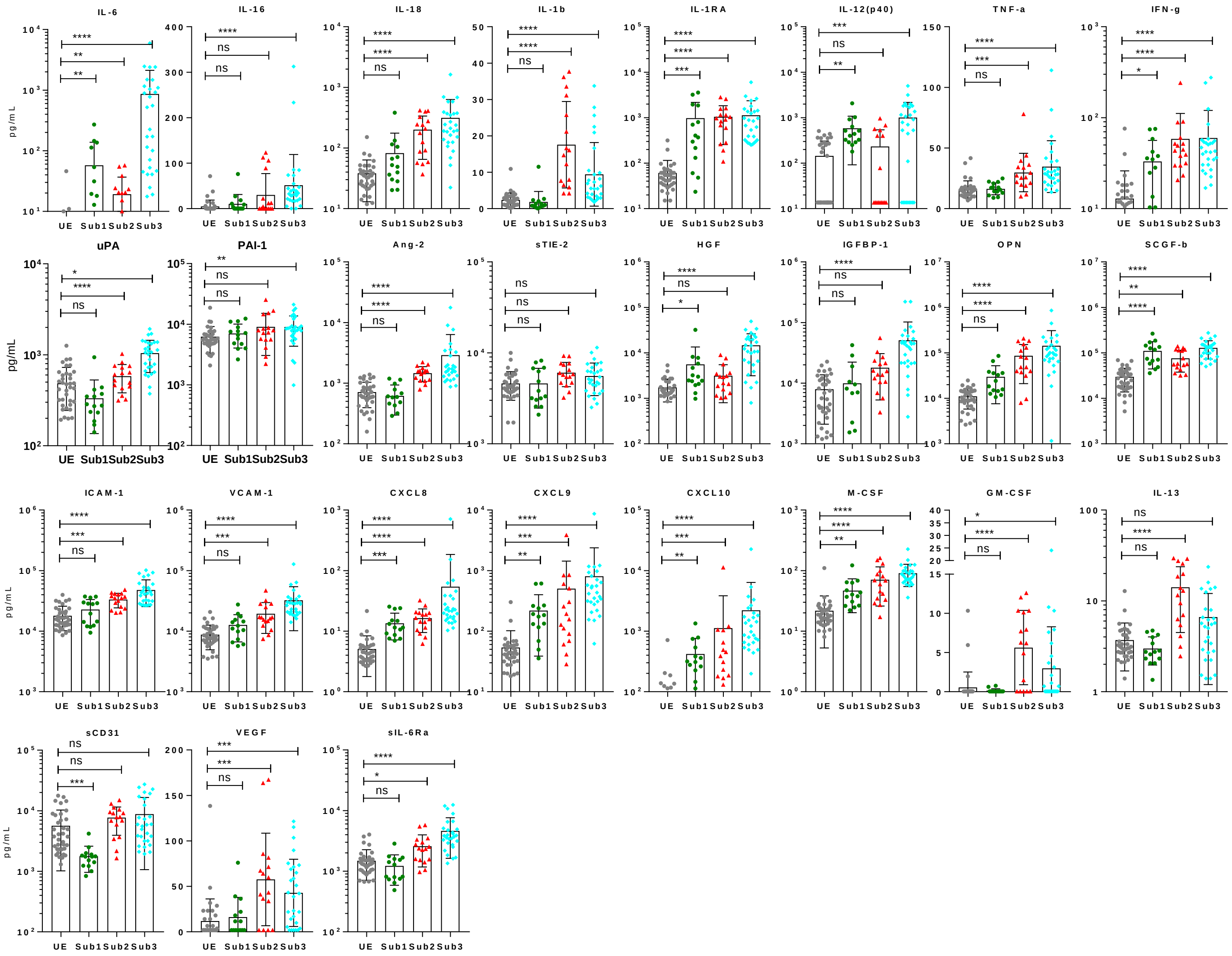

Fig. S8: Differences in plasma proteins levels among ICU subgroups compared to UE.
Luminex-based multiplex assay was used to analyze plasma protein levels in UE and ICU patients. ICU patients were classified into three subgroups (Sub1, Sub2, Sub3). Statistical analysis: Kruskal-Wallis test with test with Dunn's multiple comparison test were performed. *p < 0.05, **p<0.01, ***p<0.001, ****p<0.0001.

Table S1: Patients demographics and clinical characteristics.
Characteristics of intensive care unit patients (ICU), convalescent patients (CONV) and unexposed donors (UE). N=number of patients, n=number of samples.

**Characteristics**	**ICU cohort** N=25	**CONV cohort** N=17	**UE cohort** N=29
**Samples number n**	58	28	36
**Age** in years (Min - Max)	54,2 (18,8 - 77,2)	53,3 (18,9 – 75,5)	45,3 (21,5 – 80,9)
**Gender**			
male	44 (76%)	22 (79%)	15 (42%)
female	14 (24%)	6 (21%)	21 (58%)
**COVID risk factors/Co-morbidities**			
Chronic lung disease	3 (2)	7 (4)	0
Chronic cardiovascular disease	8 (4)	6 (4)	1 (1)
Chronic renal failure	4 (1)	1 (1)	0
Chronic liver disease	0	2 (1)	0
Cancer	6 (3)	1 (1)	0
Lymphoma	2 (1)	0	0
Pancreas	3 (1)	1 (1)	0
NSCLC	1 (1)	0	0
Diabetes	14 (8)	5 (4)	5 (3)
Adiposis	33 (15)	8 (5)	5 (3)
Hypertension	28 (14)	11 (7)	7 (5)
Pregnancy	5 (2)	2 (2)	0
Immunosuppression	8 (3)	5 (3)	0
Solid Organ Transplant	4 (1)	1 (1)	0
HIV	0	2 (1)	0
Other	4 (2)	2 (1)	0
**Clinical parameter**			
DASO (Mean, Min, Max)	25,5 (4 - 68)	120 (51 – 194)	/
CRP mg/L (Mean, Min, Max)	83,79 (2,2 - 439)	/	/
PF ratio (Mean, Min, Max)	209,3 (62 - 500)	/	/
SOFA score (Mean, Min, Max)	7,5 (0 - 18)	/	/
WHO score (Mean, Min, Max)	6 (3 - 8)	/	/
**MIS-C with COVID-19**	1 (1)	0	/
**Deceased**	5 (20%)	/	/

NSCLC: Non-small-cell lung carcinoma, DASO: Days after symptom onset, MIS-C: Multisystem Inflammatory Syndrome in Children

Table S2: Flow cytometry antibodies.
Antibodies used for flow cytometry analyses.

Antigen	Flurochrome	Manufacturer
CD3	V500	BD Bioscience
CD3	APC-H7	BD Bioscience
CD3	PerCP	BD Bioscience
CD4	PerCP	BD Bioscience
CD6	FITC	BD Bioscience
CD8	APC-H7	BD Bioscience
CD14	PE-Cy7	BD Bioscience
CD16	APC	BD Bioscience
CD19	PerCP	BD Bioscience
CD20	APC-H7	BD Bioscience
CD24	FITC	BD Bioscience
CD25	BV421	BD Bioscience
CD27	FITC	BD Bioscience
CD27	BV421	BD Bioscience
CD28	APC	BD Bioscience
CD38	APC	BD Bioscience
CD45	AF700	Biolegend
CD45	APC-H7	BD Bioscience
CD45R0	PE-Cy7	BD Bioscience
CD56	PE	BD Bioscience
CD57	BV421	BD Bioscience
CD69	FITC	BD Bioscience
CD127	AF647	BD Bioscience
CD197 (CCR7)	PE	BD Bioscience
HLA-DR	V500	BD Bioscience
IgD	PE-Cy7	BD Bioscience
